# Deforestation is the turning point for the spreading of a weedy epiphyte: an IBM approach

**DOI:** 10.1038/s41598-021-99798-5

**Published:** 2021-10-14

**Authors:** Cleber Juliano Neves Chaves, Bárbara Simões Santos Leal, Davi Rodrigo Rossatto, Uta Berger, Clarisse Palma-Silva

**Affiliations:** 1 Programa de Pós-Graduação em Ecologia e Biodiversidade, Instituto de Biociências, Universidade Estadual Paulista, Rio Claro, 13506-900 Brazil; 2 Departamento de Biologia Vegetal, Instituto de Biologia, Universidade Estadual de Campinas, Campinas, 13083-862 Brazil; 3 Departamento de Biologia, Universidade Estadual Paulista, Jaboticabal, 14884-900 Brazil; 4 Institute of Forest Growth and Computer Sciences, Technische Universität Dresden, 01737 Tharandt, Germany

**Keywords:** Computational models, Conservation biology, Invasive species, Ecological genetics, Ecological modelling, Population dynamics

## Abstract

The rapid spread of many weeds into intensely disturbed landscapes is boosted by clonal growth and self-fertilization strategies, which conversely increases the genetic structure of populations. Here, we use empirical and modeling approaches to evaluate the spreading dynamics of *Tillandsia recurvata* (L.) L. populations, a common epiphytic weed with self-reproduction and clonal growth widespread in dry forests and deforested landscapes in the American continent. We introduce the TRec model, an individual-based approach to simulate the spreading of *T. recurvata* over time and across landscapes subjected to abrupt changes in tree density with the parameters adjusted according to the empirical genetic data based on microsatellites genotypes. Simulations with this model showed that the strong spatial genetic structure observed from empirical data in *T. recurvata* can be explained by a rapid increase in abundance and gene flow followed by stabilization after ca. 25 years. TRec model’s results also indicate that deforestation is a turning point for the rapid increase in both individual abundance and gene flow among *T. recurvata* subpopulations occurring in formerly dense forests. Active reforestation can, in turn, reverse such a scenario, although with a milder intensity. The genetic-based study suggests that anthropogenic changes in landscapes may strongly affect the population dynamics of species with ‘weedy’ traits.

## Introduction

Intensive disturbances are inherent to human history and have substantial effects on natural communities^1^. Ecosystems are currently subjected to unprecedented rates of human-induced changes that are fostering the sixth mass extinction^2^. However, not all organisms respond the same way to human disturbances. While many species become extinct or migrate, others thrive in anthropogenic environments and can invade natural environments^3,4^. Indeed, human activities, such as those related to deforestation, have allowed the dispersal and reproduction of many species^4^. Weeds are opportunistic species that can evolve from introduced or native species that grow within human-transformed landscapes without being cultivated and negatively affecting both the environment and the economy^1,5,6^.

Between 50 and 80% of all invasive plant species can be classified as weeds, depending on current impacts and human perception^6^. However, native species possessing traits that pre-adapt them to establish in newly disturbed areas can also evolve into weeds^7^. In addition to landscape disturbances, propagule pressure, and individual density, the spreading rate of weeds is mainly driven by their high dispersal abilities and wide traits’ plasticity, which have enabled them to rapidly colonize large areas^5,8–11^. In particular, clonal growth and self-fertilization (hereafter referred to as ‘selfing’) are some of the most important adaptations that allow weeds to establish new populations after long-distance dispersal events^12,13^. Indeed, clonal growth and selfing favor invasiveness and weediness, even in native species, by mitigating the Allee effect of small populations and, therefore, enhance reproductive assurance and genetic transmission^14–16^. While selfing can increase species’ investment in seed production, clonal growth reduces the likelihood of genet death by sharing stochastic risk over multiple ramets^17,18^.

Despite the positive effect of clonal growth and selfing strategies on weed invasiveness, the aggregation of self-fertilized offspring and clonal individuals strongly influences the spatial genetic structure (SGS) of populations, reducing local genetic diversity and promoting inbreeding depression^18–22^. On the other hand, clonal growth may also lead to the maintenance of (once established) genetic diversity^23,24^, while continuous selfing can result in the absence of inbreeding depression as a consequence of purging of deleterious alleles over generations^16,25,26^. These effects lead to a strong population subdivision, creating a metapopulation dynamic with extinction in a site being balanced by recolonization. This dynamic may, therefore, favor selfing and clonal genotypes, due to their higher capacities of recolonization^20,27^.

Understanding local spread dynamics is essential to elucidate how native weeds opportunistically reach broad range distributions, despite their tendency to form highly aggregated populations with low genetic diversity. Individuals within populations with reduced abundance and limited seed dispersal, for instance, are usually close-related at the beginning of colonization^28^. With an increase in abundance after colonization, the general kinship often reduces due to the greater overlapping of maternal seed shadows and successive introductions of new genotypes through seed dispersal from neighbor populations^28,29^. Therefore, we expect that density-relatedness dynamics reduce the high SGS of populations of selfing and clonal species throughout colonization stages^28,30,31^.

Deforestation is another key factor for native weeds to fasten their colonization and form abundant populations into newly disturbed landscapes^32–34^. Oppositely, we also expect that coordinated actions of reforestation, or afforestation, should be an efficient strategy to control the weed spreading. Here, we integrate empirical and simulated genetic data to study the spreading dynamics of *Tillandsia recurvata* (L.) L., an epiphytic weedy bromeliad with selfing and clonal growth strategies, widely distributed in the American continent^35–37^. This species is known by several popular names (e.g., ball-moss, Jamaican ball moss, *musgo de bola*, *heno de bola*, *galinita*, and *cravo-do-mato*), which refer to the dry aspect of leaves and the intense clonal growth from leaf axils that forms a “ball-like” shape^38^ (see Fig. S1). The high abundance and dominance of *T. recurvata* in epiphytic communities constitute a characteristic feature of open landscapes from Argentina to the southern United State^38,39^ and have long attracted the interest of naturalists and ecologists^38,40–42^*.* Studies have described *T. recurvata* as the most xerophytic species among the tropical and subtropical epiphytes^43,44^, with “…*probably the greatest adaptability of any plant in the Western Hemisphere*…”^45^. Several ecological and reproductive features, such as small size, absorbent leaf scales, CAM photosynthesis, wind-dispersed seeds, the spontaneous self-pollination underpinning its tiny cleistogamous flowers, and intense clonal growth have allowed *T. recurvata* to form dense populations even on isolated trees within recently disturbed landscapes^46–48^. As an epiphytic weed, populations of *T. recurvata* develop on landscape in which each host tree performs as an isolated patch of habitat scattered in a harsh matrix^49,50^, determining that the majority of dispersed seeds of *T. recurvata* fall close to the mother and establish on the same host tree^51–53^.

The high adaptability of *T. recurvata* has fostered the species distribution in human-disturbed regions, forming large populations in urban landscapes, actively growing on fences and electric light wires^54,55^. Such great abundance and population density have led some authors to consider *T. recurvata* as an “epiphytic weed,” describing herbicides and other techniques to control its spreading^35,36^. Although *T. recurvata* cannot be classified as a parasite, studies have listed deleterious effects that massive populations may have on co-occurring epiphytes and host trees, as it competes for light with new shoots, modifies barks, and affects seedling growth^38,42^. Therefore, the reproductive biology, clonal growth, and opportunistic behavior of *T. recurvata* make this species a good model species for studying the temporal and spatial spreading dynamics of weeds.

Here, we specifically aimed to investigate (I) whether subpopulations of *T. recurvata* growing on neighbor trees of an anthropic landscape are genetically structured; as it occurs, we then tested (II) how such genetic structuring develops over time and varies in landscapes with distinct tree densities; and (III) whether abrupt human-induced changes in the landscape (deforestation or reforestation) affect the dynamics of *T. recurvata*. We hypothesize that *T. recurvata* exhibits high spatial genetic structure due to intrinsic features, such as clonal growth, selfing, and epiphytic habit. Such genetic structuring, however, might be reduced as subpopulations become denser and more connected by seed dispersal. Since this species is drought-adapted and its seeds are wind-dispersed, we also hypothesize that reductions in tree densities (i.e. deforestation) will lead to increased individual abundances and reduced genetic structure among subpopulations on trees.

To achieve our goals and properly test the outlined hypothesis we might gather numerous and continuous empirical genetic data over a long period in similar landscapes with varying tree densities, which would be intractable and very time consuming^56^. To overcome this difficulty, we adopted a modeling approach, which has been helpful to simulate spatio-temporal dynamics of species genetic structure in natural systems^57,58^. We introduce the TRec model, which takes advantage of the individual-based models (IBMs) by explicitly incorporating characteristics of each individual (e.g., life stage, size, and genotype) and simulating interactions among them and with the surroundings in multiple landscapes, which may produce complex and distinct outcomes^59^. In the study presented, we employed the combined results from microsatellite markers and the TRec model to estimate and simulate the SGS of a *T. recurvata* population scattered on neighboring trees and to understand the emergence of the observed empirical patterns of genetic variation over colonization time and across landscapes with distinct tree densities.

## Results

### Empirical genetic diversity and structure

**Figure 1:**
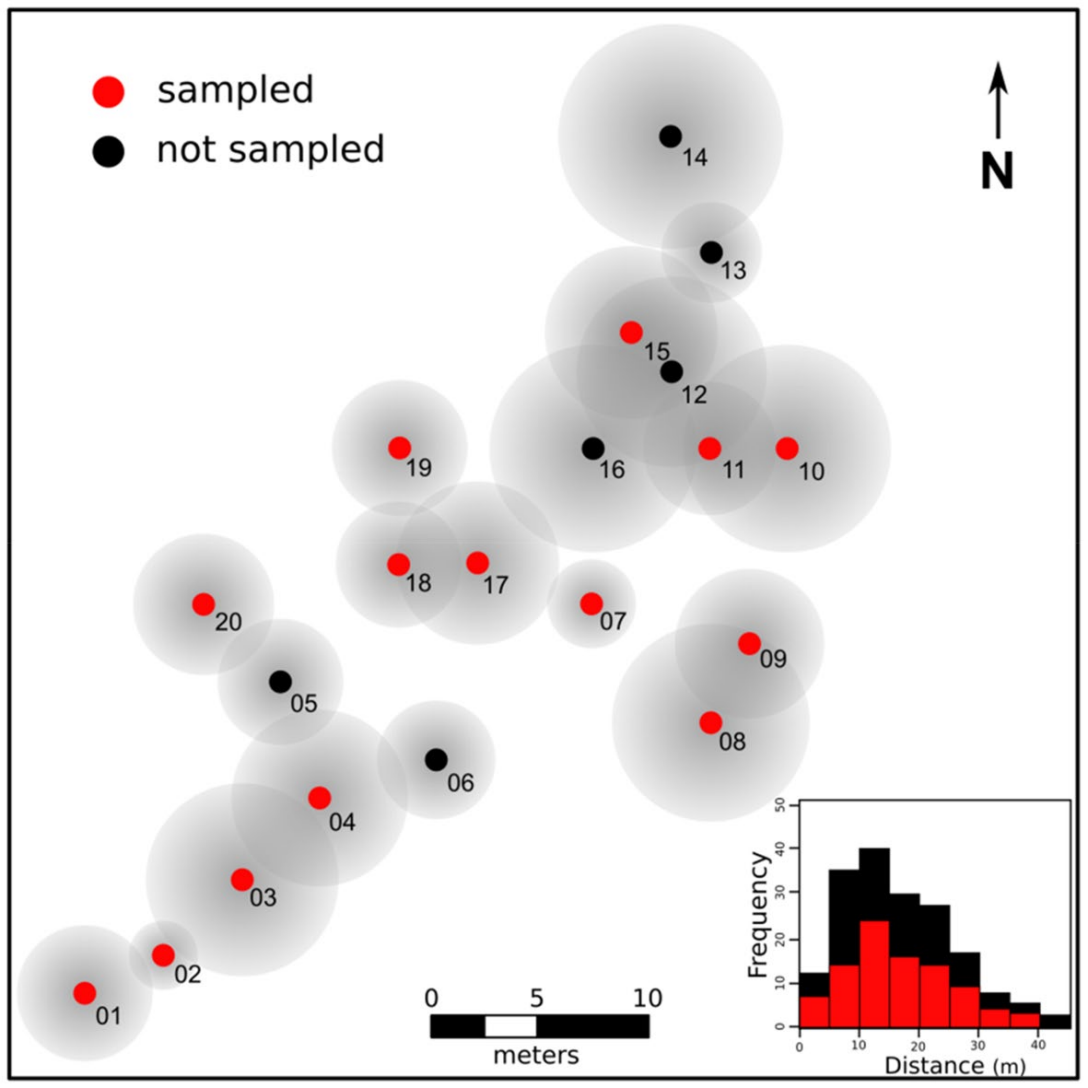
Spatial distribution and histogram of the frequency of distance classes of pairwise trees within the studied landscape. Numbers refer to IDs of trees hosting the *Tillandsia recurvata* populations analyzed. Gray circles around each dot are representations of the crown area of each tree. Red dots and bars represent, respectively, the location and frequency of distances among pairs.

The empirical data was collected in a human-transformed landscape of ca. 0.2 ha in southwest Brazil (− 21.244289° S, − 48.300486° W) composed by a grove of 20 cultivated *Handroanthus* spp. (Bignoniaceae) of similar ages (ca. 20 years) apart from each other from 2.5 to 47.5 m and surrounded by a grassland matrix (ca. 100 tree/ha; Fig. 1). Based on seven microsatellite loci genotyped in a total of 224 individuals of *T. recurvata* hosted on 14 distinct trees (considered here as subpopulations), we observed moderate levels of genetic diversity within subpopulations, despite high endogamy and generally high genetic structure. The number of alleles (A) per subpopulation ranged from 19 to 30, while allelic richness (A_R_) ranged from 2.20 to 3.54, and the number of private alleles (A_P_) was up to 2 per subpopulation (see Table S1 in Appendix S2). Expected (H_E_) and observed (H_O_) heterozygosities per subpopulation ranged from 0.27 to 0.51, and from zero to 0.20, respectively (Table S1). The inbreeding coefficients (*F*_IS_) were significant and very high in all sampled subpopulations, ranging from 0.52 to 1.0, due to significant heterozygosity deficit under Hardy–Weinberg equilibrium (Table S1). Pairwise subpopulation differentiation (*F*_ST_) ranged from 0.01 to 0.31 (Fig. S1). The Mantel test indicated a significant although weak isolation-by-distance (IBD) pattern among subpopulations only when using Edward’s genetic distances (r = 0.243; *p* = 0.032). AMOVA analysis, in turn, showed lower genetic variance among subpopulations (17.26%) than within subpopulations (82.74%) although it was significantly greater than zero (*p* < 0.001).

**Figure 2:**
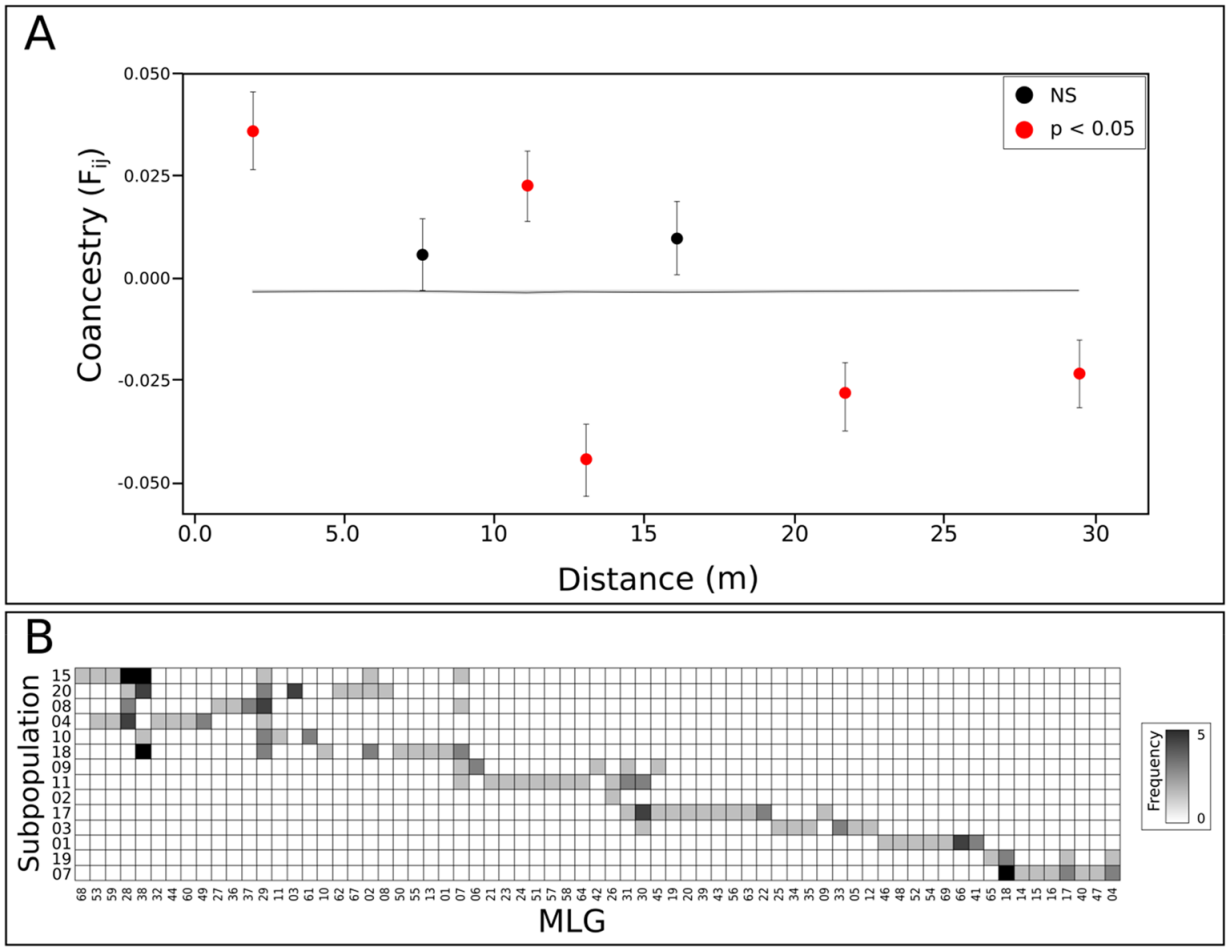
Spatial genetic structure of the empirical *Tillandsia recurvata* population. (**A**) correlogram from spatial autocorrelation analysis using the correlation coefficient Fij^59^ and seven distance classes. The line and the narrow gray area around it represent the expected kinship and 95% confidence intervals of the null hypothesis with no spatial genetic structuring. Black lines around each average F_ij_ value represent their standard errors. (**B**) Adjacency matrix showing the occurrence of each genotype (MLG) of *Tillandsia recurvata* (columns) on subpopulations corresponding to each sampled tree (rows). Grey gradient colors represent the frequency of each genotype per subpopulation.

The highest kinship (F_ij_) was detected at the smallest distance interval (up to 2.5 m), which is significantly higher than expected by chance (*p* < 0.05; Fig. 2A). Kinship coefficients peaked outside the 95% confidence interval at almost all distance classes, showing a remarkable decay at three out of four distance classes higher than ca. 13 m (Fig. 2A), and a significant spatial genetic structure (S_p_ = 0.024; *p* < 0.001). We detected turnovers of alleles and multi-locus genotype (MLG) of 0.031 and 0.537, respectively. MLGs were evenly spaced among subpopulations, generally with few shared and one distinct dominant MLG in most of the subpopulations (Fig. 2B).

### TRec model setting

**Figure 3:**
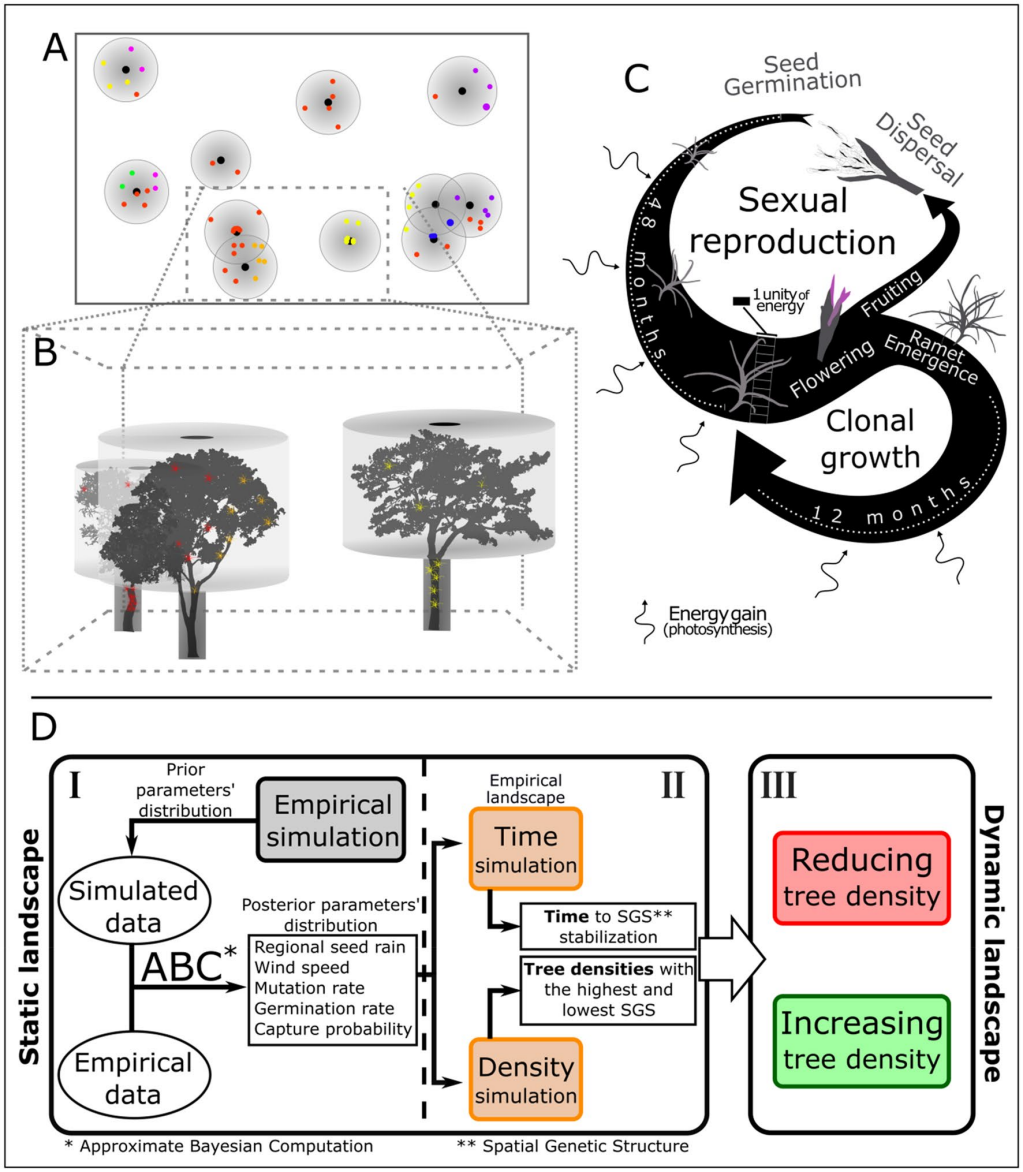
Schematic representations of the TRec model that simulates the *Tillandsia recurvata* spreading in a 0.40 ha landscape. (**A**) is a landscape representation of the model implemented in NetLogo. Points with distinct colors represent *T. recurvata* individuals of distinct genetic lineages (MLLs). Larger circles with a gradient gray color and concentric smaller black circles represent the crowns and trunks of distinct trees. (**B**) shows a 3D representation of a cut area of the landscape in (**A**). (**C**) shows a summarized representation the life cycle of *T. recurvata*. The arrow width represents the amount of energy accumulated in each life stage. Dotted lines indicate the time for maturity and reproduction. Small rectangles indicate the minimal units of energy for individual reproduction. The amount of energy accumulated is variable according to the local shading rate, affecting both sexual reproduction and clonal growth. For the TRec model, the *T. recurvata* lifespan is 72 months. (**D**) shows a flowchart diagram with the steps to adjust the TRec parameters (in white squares) to run distinct simulations (in colored rounded squares) aiming to analyze the distinct aspects of the effect of tree density on the spatial genetic structure (SGS) of *T. recurvata* populations. I: delimits the main processes for parameters estimation based on the empirical data; II: delimits the main process for estimation of time for SGS stabilization and tree densities in which the highest and lowest SGS are achieved; III: delimits the main processes to analyze the SGS variation considering dynamic landscapes.

The TRec model simulates the colonization of a landscape by multiple *T. recurvata* seeds with random multi-locus genotypes (MLG) that thrive on scattered trees (Fig. 3; see Appendix S1 for a detailed description of the model). The sensitivity test of the five varying parameters adopted in the TRec model (Fig. S3) showed that, in general, high rates of mutation and seed germination can significantly decrease the inbreeding coefficient (*F*_IS_); whereas strong winds, massive regional seed rains, and low rates of seed capturing likely reduce the SGS within *T. recurvata* populations (Fig. S2). The cross-validation showed significant correlations between real and estimated values of each parameter (*p* < 0.01). Such analysis indicated *wind speed* and *capture probability* as, respectively the most and the least accurately predicted parameters (Fig. S3). The ABC framework (‘Approximate Bayesian Computatio’^60^) successfully constricted the prior distribution of the parameters. The posterior range of these parameters, as estimated by the ABC (Table 1) were, therefore, used to perform the subsequent simulations (Fig. 3E).

**Table 1:** TRec model parameters of *Tillandsia recurvata* populations.

Parameter	Definition	Prior range (min–max)	Prior distribution	Posterior range (CI 95%)	References
Regional seed rain	Number of seeds that reach the landscape at each reproductive season	100–500 seeds/year	Uniform	88–264	Previous simulations tests
Wind speed	The magnitude of seed dispersal	1–20 units*	Uniform	13.95–21.28	Previous simulations tests
Mutation rate	Average SSR mutation-rate	10^−6^–10^−2^ (mean = 10^−4^; sd = 10^−3.5^)	Normal	10^−5.28^–10^−4.07^	^61–63^
Germination rate	Seed germination rate	0.0083–0.30	Uniform	0.162–0.297	^48,64^
Capture probability	The probability of seeds be captured in the canopy	0.01–0.30	Uniform	0.150–0.296	^65^

Prior ranges are based on literature or previous simulation tests, while posterior ranges are based on the ABC framework.

*Each wind speed unit is multiplied by five to refer to the amount of ‘landscape patches’ in the model the seeds can fly freely at each time step of the model.

#### Time and density simulations

**Figure 4:**
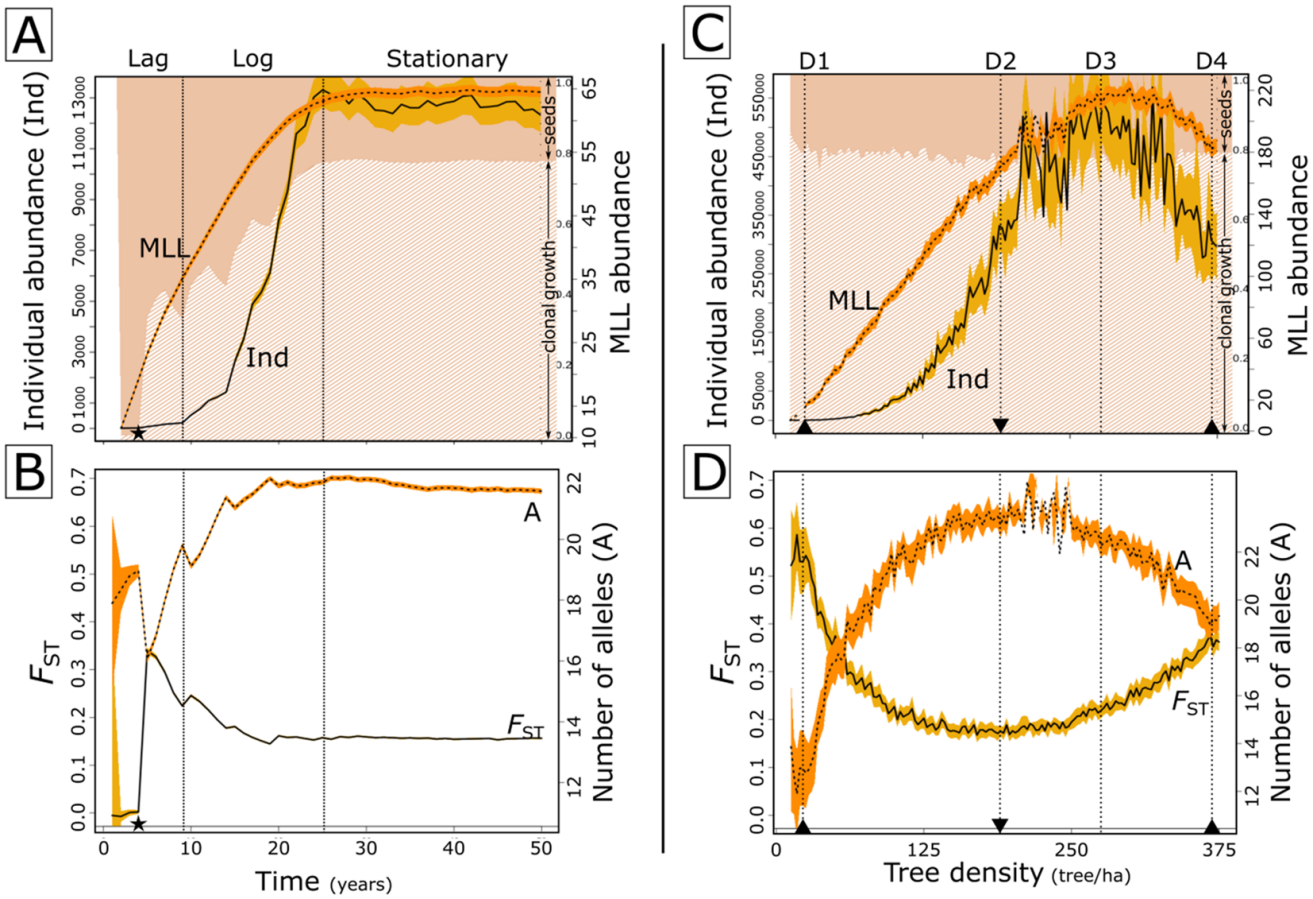
Patterns obtained from multiple simulations of the spreading of *Tillandsia recurvata* populations in the empirical static landscape (see Fig. 1) over 50 years (**A**, **B**), and in static landscapes with varying tree densities after 20 years of simulations (**C**, **D**). (**A**) and (**C**) show the abundance of individuals (solid line) and multi-locus lineages (MLLs, dotted line), as well as the clonal-growth proportion (lighter background). (**B**) and (**D**) show the pairwise subpopulation differentiation (*F*_ST_, solid line) and the average number of alleles per subpopulation (dotted line). Yellowed areas around lines represent the 95% confidence intervals of each variable in a total of 2500 (**A**, **B**) and 7000 (**C**, **D**) simulations. In (**A**) and (**B**), “Lag”, “Log”, and “Stationary” phases are delimited by vertical lines. In (**C**) and (**D**), vertical lines highlight turning points in the individual abundance and *F*_ST_ of *T. recurvata* populations (D1–D4). Stars highlight the 4th simulated year, when the first arriving seeds reach maturity. Down and up arrowheads highlight tree densities with the lowest and highest *F*_ST_, respectively.

We divided the temporal patterns of individual abundance obtained through simulations of the spreading of *T. recurvata* populations in the empirical static landscape (hereafter referred to as *time simulations*) into three distinct phases, here referred to as Lag (ca. 0 to 10 years), Log (ca. 10 to 25 years), and Stationary (higher than 25 years) phases (Figs. 4A,B and S5). During the Lag phase, the simulations showed that individuals' abundance was maintained at very low levels, while the MLL abundance grows at a linear rate up to ca. 65 MLLs, at the end of the Log phase (Figs. 4A, S5). Up to the half of the Lag phase, when the first few individuals start to clonally grow (i.e., emit new ramets) and reproduce, most of the calculated genetic statistics resulted in unstable patterns. Following this initial period, our simulations showed that the increase in the proportion of clones as well as in the aggregation of genotypes from the same MLL within each subpopulation lead the *T. recurvata* population to reach its strongest spatial genetic structuring (SGS), as evidenced by the highest values of *F*_ST_ and S_P_ (up to ca. 0.35 and 0.01, respectively), and the lowest mean number of alleles per locus and subpopulation (A; ca. 16) and expected heterozygosity (H_E_; ca. 0.53). The simulations followed to the Log phase as individuals from the same MLL (commonly with the same genotype) started to disperse seeds to other trees, leading to a boom in individuals abundance and a subpopulation differentiation decay (Fig. 4B). At this phase, the individual abundance rapidly multiplied, mostly by clonal growth, reaching a peak of ca. 12,500 individuals (ca. 192 individuals/MLL; Figs. 4A, S5), increasing the gene flow among subpopulations and leading to a drop in the SGS (i.e. reducing F_ST_, S_P_, and MLG and MLL turnover while increasing A and H_E_). As the individual abundance saturated, at the Stationary phase, all measured variables from simulated genetic data suffered only small variations (Fig. 4B).

From the simulations with distinct tree densities (hereafter referred to as *density simulations)*, we highlighted four key values of tree density in ascending order (see D1 to D4 in Figs. 4C,D, S5) that denote turning points in the individual abundance and SGS of *T. recurvata* populations. Landscapes with ca. 20 trees/ha (D1) showed the lowest individual abundance, A, and H_E_, and the strongest SGS (i.e. the highest *F*_ST_, Sp, and MLG- and MLL-turnover). Conversely, landscapes with ca. 190 trees/ha (D2) and 275 trees/ha (D3) showed, respectively, the lowest SGS and the highest individual abundance (ca. 550,000 individuals from 220 MLLs). Finally, landscapes with increasing tree densities from 275 (D3) to 375 trees/ha (D4) tended to gradually hold fewer individuals and MLLs as long as the SGS enhances.

#### Dynamic simulations

**Figure 5:**
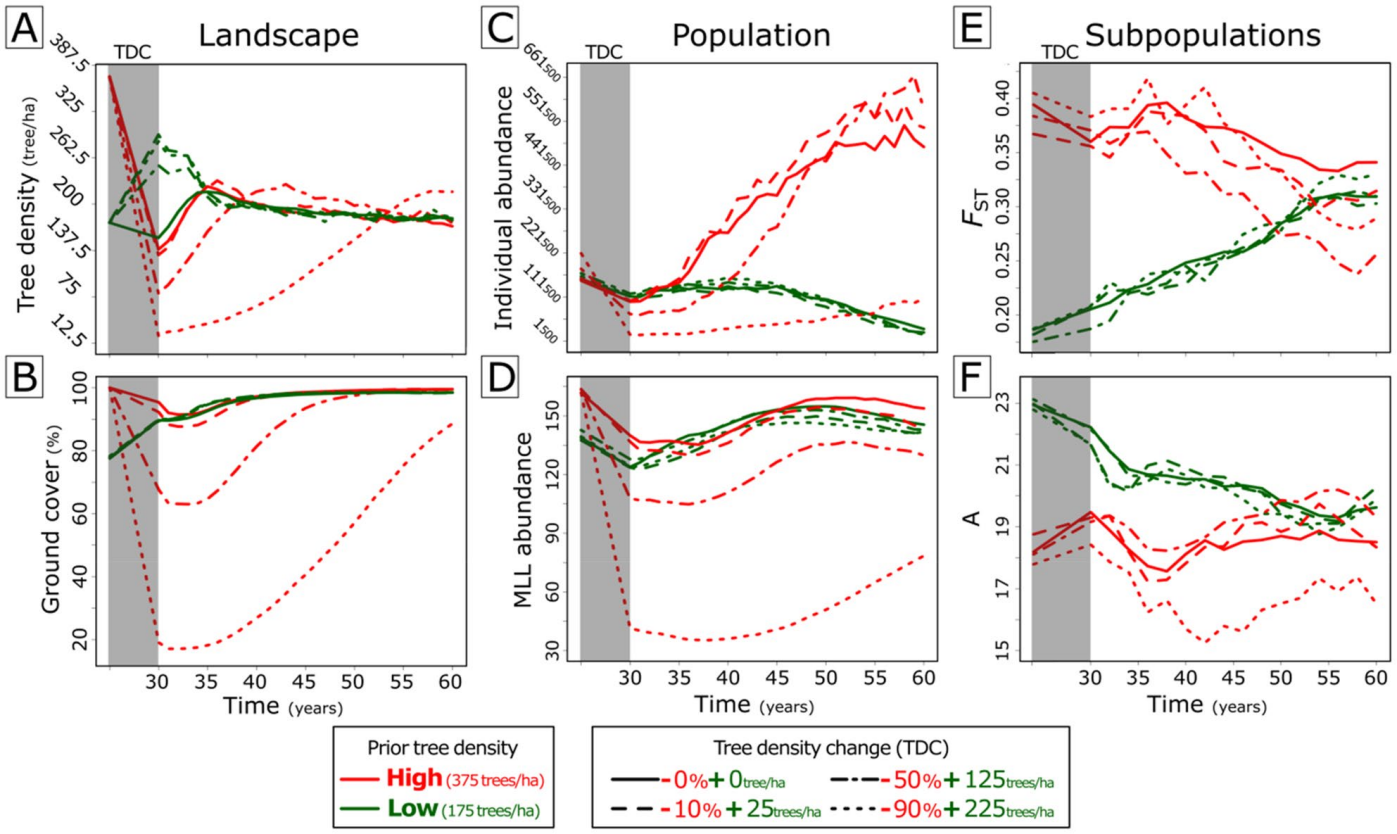
Temporal patterns obtained from multiple simulations of the spreading of *Tillandsia recurvata* populations in dynamic landscapes (emulating tree growth, regeneration, and self-thinning of natural forests) with prior high and low tree densities after abrupt reductions (lines in red) or increments (lines in green) in tree densities (shaded areas). The first 30 simulated years, not shown here, correspond to the simulations in static landscapes presenting the lowest and highest F_ST_ (considering high tree density conditions) showed in Fig. 4C,D. From the first to the last column, the figures show 30-years of posterior temporal variation in landscapes’ tree density (**A**) and ground cover (i.e. the relative number of simulated landscape patches not covered by tree trunks or crowns; (**B**); in the *T. recurvata*’s population abundance of individuals (**C**) and multi-locus lineages (MLLs; (**D**); and in subpopulation’ differentiation (*F*_ST_; **E**) and the average number of alleles (**A**; **F**). Lines represent the averages of each represented variable in 2000 simulations. Solid, dashed, dotted-dashed, and dotted lines represent, respectively, absent, low, intermediate, and high anthropogenic changes in tree density (TDC).

From the simulations with dynamic landscapes (i.e. allowing tree growth, regeneration, self-thinning), we observed that removing up to 90% of the trees from a high-tree density landscape led to 80% less ground coverage, while the addition of new trees to lower-tree density landscapes led to smaller changes (ca. 10%) in the ground cover (see shadowed areas in Fig. 5). Our model showed that deforestation resulted in the loss of up to 200,000 individuals and 120 MLLs of *T. recurvata* populations (Fig. 5C,D) but to smaller changes in terms of genetic diversity and structure (i.e. A and *F*_ST_; Fig. 5E,F). The further effects of tree growth, regeneration, and self-thinning, in turn, unfolded in the convergence of all simulated scenarios to tree densities of ca. 180 trees/ha (Fig. 5A). The simulations also showed a tendency of convergence in the proportion of ground cover (ca. 100%; Fig. 5B) and MLL abundance of *T. recurvata* populations (ca. 140 MLLs; Fig. 5D) among such distinct scenarios after the first 60 years following the tree density changes. Populations from deforested and reforested landscapes, however, showed distinct patterns of individual abundance (Fig. 5C). While *T. recurvata* populations from deforested landscapes increased up to ca. 600,000 individuals between the 30th and the 60th year, populations from reforested landscapes shrank up to 35% of individuals from the 45th year onwards. The differences in the number of individuals per MLL (Fig. S6) resulted in reducing SGS over time in populations from deforested landscapes and an increasing SGS over time in populations from reforested landscapes (Fig. 5E). Compared to the low and moderate tree density changes, the scenario with a massive loss of 90% of trees led to a particularly weaker increment of individuals (Fig. 5C). Such increment resulted in individual abundances closer to that observed in reforested landscapes, but with a lower mean number of alleles per subpopulation (A; Fig. 5F).

## Discussion

In this study, we integrated empirical and simulated genetic data to provide insights into the spreading of the epiphytic weed *Tillandsia recurvata*, which is widespread into human-transformed landscapes. Our empirical data reveals a relatively high spatial genetic structure (SGS; i.e. low gene flow) in a small landscape (c.a. 0.2 ha) with low tree density and opportunistically colonized by a massive population of this species. Each tree in this system functions as habitat units, forming distinct subpopulations of *T. recurvata* that group specific sets of multi-locus genotypes (MLG). The data simulated by our TRec model indicate that the highest levels of SGS in *T. recurvata* occur at the very beginning of the colonization process and under extremely low or high tree densities. As expected, lower SGS occur at landscapes in which seed dispersal is limited neither by long distances among spaced trees nor by the low carry capacity of wind among dense canopies. The TRec model also shows that deforestation is a turning point for the opportunistic thriving of *T. recurvata* populations. Our results contribute to the understanding of the dynamics of species invasiveness and landscape invasibility and also on the consequences of human-induced changes for the spreading of opportunistic species, as we discuss below.

### The patchy distribution of a *T. recurvata *population

Our empirical study in an anthropic landscape showed a relatively high to moderate SGS for a population of *T. recurvata* at a fine-scale (ca. 2000 m^2^), as indicated by the significant S_p_ statistics and high subpopulation differentiation. Such SGS likely arises as an outcome of the breeding system and life form of *T. recurvata*, which limits local gene flow, resulting in a significant isolation-by-distance, persistence of private alleles in six out of 14 studied subpopulations, as well as high MLGs turnover. Indeed, fine-scale SGS often results from limited gene flow^66,67^, and other studies have reported similar genetic patterns for plants with, self-reproduction^68,69^, clonal growth^69,70^, epiphytic habit^51,52,71^, fast-growth^72,73^, and high-density populations^66,74^. Our results indicate, therefore, that specific habits and breeding systems can foster the structuring of populations, even for opportunistic and widespread plants.

The few MLGs shared among subpopulations indicate that the *T. recurvata* spreading is gradually conducted by several multi-locus lineages (MLLs) following a phalanx pattern, rather than by a single leading MLL^40^. The relatively low S_P_ observed for a selfing species^66^, in turn, seems to be related to its dense populations^66,74^. These results suggest that multiple and dense sources of *T. recurvata* propagules from the surrounding areas are responsible for the massively spreading over the studied landscape. Under such conditions, self-reproduction and clonal growth^46–48^ may lead to a partial, and perhaps transitory, isolation among subpopulations^67^. Beyond the effect of the breeding system and life form, the relatively high SGS of *T. recurvata* population may also result from founder events on each host tree^40,67^. Nevertheless, the genetic drift resulting from consecutive founder events, as well as the increasing number of seeds released from large subpopulations, may intensify the metapopulation dynamics of successive local extinctions and recolonizations, reducing the genetic structure of the population over time^12,13,27^.

### Temporal patterns in the opportunistic spreading of *T. recurvata*

The temporal dynamics of the simulated *T. recurvata* population resembles the diffusion models of weedy species invasion in newly disturbed landscapes, with sequential Lag, Log, and Stationary phases^75^. During the Lag phase (up to the 10th year), only a few *T. recurvata* individuals successfully establish in the landscape, despite the constant input of new genetic lineages (MLLs) per reproductive season. At this stage, as clonal growth continues and the proportion of clones increases, leading to aggregated individuals holding the same genotypes within each subpopulation, the population reaches the highest genetic structure. The reproduction start of such large groups of genetically related individuals leads to a boom in the overall individual abundance, part of the newly produced seeds is exchanged among subpopulations and causes a gradual decrease in the genetic structure, as observed in other empirical studies^47,66^. It is notorious, at this point, the impact of clonal-growth in *T. recurvata* spreading, overcoming the demographic stochasticity and the Allee effect^10,76,77^, and leading the population to the following Log phase (10th to 25th year).

The rapid increase in individual abundance during the Log phase drives the interchange of seeds, and therefore MLGs, among subpopulations, leading to an overall reduction in subpopulation differentiation and genetic structuring. However, the saturation of the static system limits the establishment of a higher number of individuals and MLLs, maintaining all calculated statistics at a steady level after the 25th year (the Stationary phase). Such increment and further stabilization of gene flow at relatively high levels indicate a potential role of *T. recurvata* populations as a source of seeds for supporting the species spread into newly disturbed areas after ca. 25 years of colonization. Notwithstanding the specific characteristics of each species, this temporal pattern is typical for opportunistic organisms growing under optimal conditions^75^. As observed for *T. recurvata*, a fast increase and stabilization in the individual abundance of populations and communities are expected for static systems^78,79^. However, given their xerophytic preference, distinct patterns in the spreading of *T. recurvata* populations can came across in landscapes with varying tree densities, as we see in the following section.

### Effects of tree density on *T. recurvata *spreading

Our model indicates that the highest gene flow (i.e. the lowest SGS) among *T. recurvata* subpopulations occur in landscapes with ca. 190 trees/ha, which likely represents the ideal condition for the species spreading. Landscapes with fewer trees tend to harbor fewer individuals of *T. recurvata*, from which the seeds have to cross longer distances that likely prevent gene flow. On the other hand, landscapes with more than 190 trees/ha counterbalance the effect of increasing habitat amount for epiphytic hosting^80,81^ with the intensification of shading and wind friction for seed dispersal^82–84^. Indeed, as we observed here, studies implementing IBM approaches for distinct biological systems have shown that the abundance and genetic structuring of populations result from the interplay between habitat amount and resistance to gene flow across landscapes^57,58^. For *T. recurvata*, unlike other epiphytes^80,85^, the heavy overlapping of tree crowns in dense forests likely reduces the species' fitness, resulting in small, transient, and isolated populations constrained to the driest and upper canopy layers^37,80,86,87^. Further studies incorporating the genetic component of fitness into the models can give more insights into the colonization process and the invasiveness of this opportunistic species. The increasing number of genome-wide markers generated by high-throughput sequencing, as well as the constant development of computational methods, will also allow future investigation on the relative importance of natural selection on the genetic structure of such species.

### Changing landscapes and opportunistic spreading of *T. recurvata*

The combined effect of abrupt reductions in tree density and natural self-thinning have enabled the opportunistic thriving of *T. recurvata* populations in formerly densely forested landscapes. Indeed, studies have suggested that conditions found in dense forests typically prevent the colonization and development of this species, given the limited carrying capacity of wind for seed dispersal^80–84^. Using our model approach, we observed an overgrowth of *T. recurvata* populations in recovering landscapes that suffered low to moderate tree removal, with a significant increase in gene flow. The massive deforestation (i.e. 90% of tree removal), in contrast, reduces drastically the number of available host trees, leading, as expected, to smaller populations of *T. recurvata* and increasing gene flow among subpopulations as the forest regenerates.

Reforestation, in turn, slowly leads *T. recurvata* populations in the opposite direction. In our model, the presence of only 20% of uncovered ground (i.e. spaces uncovered by tree trunks or crowns and, thus, able to receive new trees) under lower-tree densities constrained the active increment of trees, reducing the differences among the distinct levels of reforestation. Despite the milder effects in comparison to deforestation, the active increase in tree density and the continuous growth of the former trees likely reduce the abundance of *T. recurvata* and the gene flow among subpopulations. The larger and older trees in reforested landscapes, compared to the smaller and younger trees in landscapes following deforestation, could play a major role in increasing shading and reducing the carrying capacity of wind for seed dispersal. Therefore, our results indicate that the control of *T. recurvata* spreading depends not only on the tree density of landscapes but also on the stage of forest recovery. In other words, the positive effects of controlling weeds with posterior reforestation, besides costly, can be much more time-consuming than just avoiding deforestation.

## Conclusion

Opportunistic species provide an excellent system to examine the process of successful colonization in novel environments^7^. Here, we demonstrate that ‘weedy’ traits, such as selfing and clonal growth^13,18,19^, may lead populations to distinct outcomes depending on the landscape conditions. In some landscapes, such traits undermine gene flow and genetic diversity, potentially increasing inbreeding in populations^18–22^, but under ideal conditions, they can also mitigate the Allee effect by assuring the genet survival, seed dispersal, and genetic transmission^14–16^. Our study also shows that anthropogenic deforestation is the turning point to increase the invasibility of landscapes, enabling the rapid thrive and spreading of *T. recurvata* especially onto recovering landscapes, where smaller trees produce reduced cover on once heavily shaded ground by larger trees. As have been shown by other studies^88–90^, our findings reveal that reforestation has milder and slower effects on mitigating the impact of massive tree removal on the spreading of opportunistic species, highlighting the importance of maintaining forests instead of investing in restorations. On the other hand, landscape features determined by shading, as well as growth, reproduction, and competitiveness among trees likely lead to alternative outcomes for epiphytes. Testing the impact of distinct environmental conditions (e.g. soil, climate, etc.) on the assembling of tree groups would be a wise step toward a more global understanding of invasion ecology.

## Methods

### Empirical genetic data

The empirical study took place in a human-transformed landscape of ca. 0.2 ha in southwest Brazil (− 21.244289° S, − 48.300486° W) composed of a grove of 20 cultivated *Handroanthus* spp. (Bignoniaceae) of similar ages (ca. 20 years) apart from each other from 2.5 to 47.5 m and surrounded by a grassland matrix (ca. 100 tree/ha; Fig. 1). As it is common in many human-transformed landscapes, the empirical landscape results from the complete deforestation followed by a landscaping procedure that prevents non-planted specimens to grow amongst introduced trees on which a massive *T. recurvata* population has colonized.

We sampled a total of 224 individuals of *T. recurvata* growing on 14 neighboring trees (16 individuals per tree; hereafter referred to as “subpopulations”) and extracted the total genomic DNA from leaf samples according to Tel-Zur et al.^91^. The collection of plant material complied with national guidelines. Permits to collect in conservation units were granted by COTEC (Technical and Scientific Committee of the Forestry Institute, São Paulo, Brazil; permission number 006.221/2014) and SISBIO (Biodiversity Information and Authorization System, Brazil, permission number 44443–1). We characterized the multi-locus genotype (MLG) of each individual using seven microsatellite loci designed for other species and cross-amplified them as described by Chaves et al.^92^. We avoided sampling clones by collecting individuals at distinct branches of each host tree. Indeed, clonal individuals of *T. recurvata* (i.e., ramets) can be easily distinguished because they are formed on leaf axils and remain attached, conferring the typical "ball" shape of the species^38^.

To describe the genetic diversity within each *T. recurvata* subpopulation, we calculated the number of alleles (A), allelic richness (A_R_), the private number of alleles (A_P_), expected (H_E_) and observed heterozygosity (H_O_), and inbreeding coefficient (*F*_IS_) and tested for departures from Hardy–Weinberg equilibrium using the R statistical package diveRsity^93^. Furthermore, we tested whether subpopulations were isolated-by-distance using a Mantel test with Slatkin’s linearized *F*_ST_ (*F*_ST_/(1 − *F*_ST_))^94^, Nei’s^95^, Edward’s^96^, and Reynold’s^97^ pairwise genetic distances and the logarithm of geographical distance using the R statistical packages poppr^98^ and pegas^99^. We also measured pairwise subpopulation differentiation (*F*_ST_) and tested the hierarchical partition of genetic variance among and within sampled subpopulations by performing an analysis of molecular variance (AMOVA^100^), using the R statistical package poppr^98^.

To investigate the spatial genetic structure (SGS) within the *T. recurvata* population sampled, we tested whether the distance among *T. recurvata* individuals (disregarding tree crowns) affects their relatedness. For this, we estimated the kinship coefficient (F_ij_^101^) between all pairs of individuals sampled within seven distance classes, each comprising the same number of pairs, using the R package RClone^102^. To quantify the SGS under this approach, we calculated the S_p_ statistics (i.e. the ratio between the kinship decay by increasing geographic distances and the mean kinship within the first interval distance^66^) and tested the statistical significance of the mean kinship coefficient values in each distance class (confidence interval: 95%) by randomly shuffle (1000 ×) individual locations^66^. We also quantified the partitioning of the genetic composition of the whole population by calculating the turnover of alleles and multi-locus genotypes among subpopulations (MLG^103,104^), using the R statistical package vegetarian^105^.

### The TRec model

We implemented the TRec model in NetLogo 6.0.1^106^ and outlined it following the ODD (Overview, Design concepts, Details) protocol formulated by Grimm et al.^107,108^ (see Appendix S1 in Supporting Information). The model simulates a landscape composed of multiple 0.01 m^2^ patches of soil with scattered trees that are colonized by multiple seeds of *T. recurvata* with distinct MLGs (based on empirical alleles). Such seeds annually reach the landscape from outside (hereafter referred to as ‘regional seeds’) by wind dispersal and generate mature individuals that reproduce by self-fertilization and grow by producing new ramets (clonal growth) on trees within the landscape (Fig. 3). The wind speed is quantified in correspondence with the total distance (in units of ‘landscape patches’) in which seeds are carried at each time step. The colonization dynamics of the model is primarily based on the ‘energy budget’ of *T. recurvata*, which quantifies, under a simplified manner, the amount of energy each individual takes up by photosynthesis and expends during its life cycle through metabolism, growth, and reproduction (including seed dispersal and clonal growth), depending on the shading rate and competition of its attachment site (Fig. 3). The energy budget approach is based on the Dynamic Energy Budget theory of Kooijman^109^ and has been adopted in IBMs to generally describe how individuals acquire and expend energy with simple and sufficient realism^110–112^. Therefore, at each time step, the TRec model update the amount of energy of every individual simulated. The survival as well as the capacity of each *T. recurvata* individual to grow and reproduce depends on the amount of energy stocked up until the reproductive season (Fig. 3). In the TRec model, mature individuals (i.e. older than 48 months) with a energy budget with at least 10 units will grow clonally and produce and disperse new seeds in the reproductive season (Fig. 3). *T. recurvata* individuals can reproduce only once and die when they reach their lifespan (72 months) or whether they maintain a net-zero gain of energy, with expenses surpassing gain (i.e. energy equals to 0 units). For the TRec model, we assume that each seed that successfully develops and thrives in the landscape forms a multi-locus lineage (MLL) of multiple descendants, which can be traced back to the ancestor. Each new seed produced within the landscape can also be wind-dispersed by chance from the mother to other trees, but new ramets are always attached to the same host tree. Each MLL often groups multiple MLGs due to heterozygous loci (according to the heterozygosity observed in the empirical study). Another source of genetic variation within MLLs is simulated by a varying mutation rate (from 10^–2^ to 10^–6^) that simulates DNA replication slippages of SSR loci.

The simulation process using the TRec model was comprised of three subsequent phases (Fig. 3E): (I) parameters estimation based on the empirical data using approximate bayesian computation (ABC); (II) estimation of time for SGS stabilization and densities in which the highest and lowest SGS is achieved; and (III) analysis of the effect of gradual and abrupt changes of tree densities in a dynamic landscape. For phase I (Fig. 3E), we simulated 250,000 microsatellite datasets generated by random combinations of five parameters with a priori estimates according to general assumptions or previous knowledge on the species (see Table 1). We then performed a Global Sensitivity Analysis to test whether summary statistics—i.e., the mean number of alleles (K), mean range of allele size (R), mean expected heterozygosity (H_E_), mean inbreeding coefficient (*F*_IS_), global subpopulation differentiation (*F*_ST_), and mean modified Garza-Williamson statistic^113,114^—as well as *T. recurvata* abundance and the total number of MLL, are differently affected by the variation of each parameter. To improve the TRec model accuracy, we estimated posterior distributions of the model parameters in an ABC framework (‘Approximate Bayesian Computation’^60^). We employed the network algorithm implemented in the ‘abc’ R package^115^ to estimate such distribution based on 0.1% retained simulations that most resembled the empirical SSR data according to the six above-mentioned summary statistics (i.e. K, R, H_E_, F_IS_, F_ST_, and NGW).

#### Time and density simulations

For the second phase of the simulation process (see Fig. 3E), we used the estimated posterior range of each Trec parameter (see Table 1) to estimate the time needed for the stabilization of the spatial genetic structure (SGS) of *T. recurvata* populations (hereafter referred to as '*time simulation*’) and the tree densities values for which the lowest and highest SGS values are achieved (hereafter referred to as ‘*density simulation*'). To reduce the bias effect inherent to dynamic landscapes (e.g. with growing and falling trees), we performed both *time* and *density* simulations in static landscapes. We performed 2500*-time simulations* that emulate 50 years of spreading of *T. recurvata* individuals on the empirical landscape representation (Fig. 1), and 5000 *density simulations* of *T. recurvata* populations in ca. 0.40 hectares landscapes with five to 150 trees (maximum amount of trees that occupy the landscape) with random heights and same crown sizes. Yearly (for the *time simulations*) and after 30 years (for the *density simulations*), we recorded the individual and MLL abundances, as well as the MLG, MLL, and ramets’ origins (from clonal growth or sexual reproduction; hereafter referred to as ‘*clones*’ and ‘*seeds*’ individuals) of up to 15 random *T. recurvata* individuals established on each host tree representation of the empirical study (for the *time simulations*) or on up to 15 random trees for *density simulations* (for the *density simulations*). Based on the MLGs of each simulation, we calculated the average number of alleles (A), the expected heterozygosity (H_E_), the inbreeding coefficient (*F*_IS_), and the global subpopulation differentiation (*F*_ST_), using the arlsumstat console version of Arlequin 3.1; the MLG and MLL turnovers among subpopulations (T/O_MLG_ and T/O_MLL_), using the R package vegetarian^104,105^; and the S_p_ statistics considering seven distance classes as in the empirical study^66^ using the R package RClone^102^.

#### Dynamic simulations

To emulate the effect of human-induced changes of tree density on the SGS of *T. recurvata* populations (the phase III in Fig. 3E), the hereafter referred to as ‘*dynamic simulations*’ add distinct levels of changes that actively reduce or increase tree density mimicking either deforestation or reforestation. For this, the *dynamic simulations* firstly simulated 30 years (when the SGS stabilizes) of *T. recurvata* spreading over static landscapes with low (175 trees/ha; when *T. recurvata* populations showed the lowest SGS) and high tree densities (375 trees/ha; when *T. recurvata* populations showed the highest SGS in landscapes with high tree densities). After these first 30 years, the model emulates distinct levels of anthropogenic reductions (0, 10, 50, or 90% of the prior tree density) and increments in tree density (by adding 0, 25, 125, or 225 trees/ha) in high and low tree density landscapes, respectively. Thereafter, we yearly calculated individual and MLL abundances, A, H_E_, and *F*_ST_ using the arlsumstat console version of Arlequin 3.1 over 30 additional years in landscapes allowing tree density variation as the result of tree-growth (an average DBH increase of 0.13 cm/year^116^), self-thinning (according to the Yoda’s law), and natural regeneration (a fixed rate at 15%; for further information, please see the ODD in the Supporting Information). Here, as in the *time* and *density simulations*, we also included the estimated posterior range of the IBM parameters (see Table 1).

## Supplementary Information

Supplementary Information 1.

Supplementary Information 2.

## Data Availability

The microsatellite data generated in this study, as well as all scripts used in the simulations, are stored at the FigShare repository [10.6084/m9.figshare.12797846].
