# Supplementary material for: Deforestation is the turning point for the spreading of a weedy epiphyte: an IBM approach
Source: Sci Rep. 2021 Oct 14;11:20397. doi: 10.1038/s41598-021-99798-5 (PMC8516858; doi:10.1038/s41598-021-99798-5)
Supplement: Supplementary file 1 — Supplementary Information 1. [file 41598_2021_99798_MOESM1_ESM.docx]

***TRec model ODD (overview, design concepts, details) protocol***

1. *Purpose*

TRec model simulates the colonization of a group of trees by populations of one of the most abundant and distributed atmospheric bromeliad species, the ‘ball-shaped’ *Tillandsia recurvata* (L.) L. (Fig. S1 in Appendix 2), with distinct multilocus genotypes (MLG). This epiphytic bromeliad occurs in the American continent, from Argentina to the south of the U.S.A. (Smith and Downs 1977; GBIF 2017), and can form populations of hundreds of individuals in trees and shrubs of native and anthropic environments (e.g. Birge 1911; McWilliams 1992; Flores-Palacios et al., 2015). Despite its huge distribution, *T. recurvata* can produce just a few dozens of seeds in each reproductive season (many of them sterile) and has a low germination rate, probably due to the absence of endosperm (Bernal et al. 2005; Chilpa-Galván et al. 2018). On the other hand, the species has a rapid life cycle, cleistogamous flowers with autonomous self-fertilization, wind-dispersed seeds, and the ability for intense clonal reproduction to group dozens of ramets that stay linked together in a ball-shaped genet producing their seeds in each reproductive season (Soltis et al. 1987; Smith et al. 1989; Orozco-Ibarrola et al. 2015; Chilpa-Galván et al. 2018).

With the TRec model, we want to understand the emergence of the observed patterns obtained by our empirical study with 14 sampled trees from a grove of 20 individuals of *Handroanthus* spp. (Bignoniaceae) of similar ages (ca. 20 years) and growing from 2.5 to 47.5m from each other, surrounded by a grassland matrix (ca. 100 tree/ha; Fig. 1 in the main text). We also aim to transcend the emerged pattern by (i) tracing the past and forecasting the future of the empirical *T. recurvata* population; (ii) projecting the emerged pattern to simulated landscapes with distinct tree densities; (iii) and testing the effect of gradual and abrupt changes in tree landscape on *T. recurvata* populations. It is hypothesized that the observed spatial genetic structure (SGS) in the real landscape is due to the higher probability of seeds being attached to the same tree where they were produced, compared to the probability to attach to other trees, forming populations with low genetic diversity. However, these results could have distinct outcomes throughout colonization time and in landscapes with different tree densities (Fig. 1). At the very beginning of *T. recurvata* spreading over a new landscape, SGS should be the strongest, but it reduces according to the offspring of the first arriving seeds spread over the trees and become a single large population (Fig. 1A). SGS may also change under distinct tree densities. Overlapping tree crowns in areas with intermediate tree densities, for instance, could act as a bridge for the seed capturing, but the massive overlap among these crowns, under high tree densities, could acts inversely, reducing the local wind speed and shading, which hamper seed dispersal, increase the boundary layer and reduce photosynthesis rate (Fig. 1B). Since *T. recurvata* is a drought-adapted species (e.g. Benzing 2000; Benzing 2012), these environmental conditions could lead to a reduction in the species' population and increase its mortality. Conversely, the seed capturing in areas with very low tree densities could also be hampered, since there is less substrate for the attachment of seeds, also increasing SGS (Fig. 1B). Therefore, abrupt changes in tree density (e.g. deforestation or reforestation) may lead to strong changes in the SGS level of *T. recurvata* populations. At the same time, all SGS dynamics will also depend on the mutation rate of *T. recurvata* markers and on (i) the wind power to carry the seeds of *T. recurvata*; (ii) the number of seeds from the regional seed rain that reaches the landscape at each year; (iii) the germination rate of *T. recurvata* seeds; and (iv) the probability of seeds captured by potential host trees.


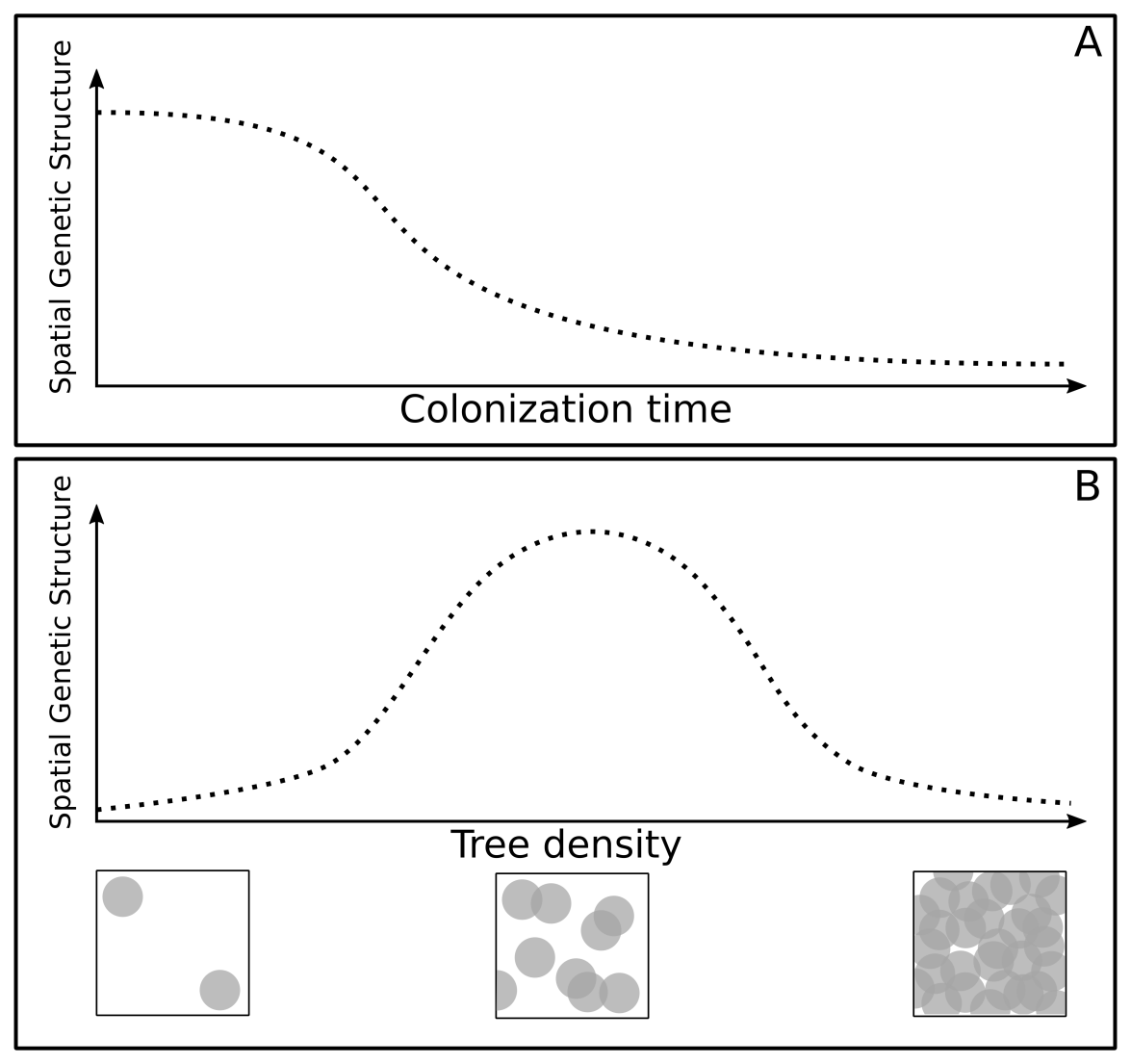


**Figure 1.** Schematic representation of the relationship of the spatial genetic structure of *Tillandsia recurvata* population with the colonization time of a landscape (A), and with the tree density of landscapes. The squares in B represent landscapes with distinct numbers of trees (gray circles) at a top view looking down.

1. *Entities, state variables, and scales*

The TRec model follows the growth and spreading of a *T. recurvata* population on trees scattered in a two-dimensional landscape at monthly intervals. The landscape area is comprised of multiple 0.01 m^2^ patches of soil and can be a representation of both an ‘empirical’ or ‘simulated landscape’. The ‘empirical landscape’ option has trees with predefined sizes and positions, similar to our empirical study site with *T. recurvata* populations (Table 1), scattered in a rectangular area of ca. 0.2 ha. The ‘simulated landscape’ option, in turn, has randomly distributed trees scattered in a squared area of ca. 0.40 ha., with the same length as the ‘empirical landscape’ option. Each tree in the landscape has distinct sizes (in meters), which are defined according to the trunk height, crown height, crown area, and diameter at breast height (DBH; see Table 1). Individuals of *T. recurvata* are characterized by their age (in months), lifespan (in months), the height of attaching on the host tree (in meters), origin ("seed" or "clonal growth”), location on the host tree (“canopy” or “trunk”), genotype (comprising two alleles of each of the seven SSR markers used in the empirical study), and energy (in arbitrary units). To simulate the effect of shading levels and competition on the fitness of each atmospheric bromeliad, we simulated their energy budget, which quantifies, in a simplified approach, the amount of energy each individual takes up by photosynthesis and expends during its life cycle through metabolism, growth, and reproduction (including seed dispersal and clonal growth), depending on the shading rate of its attachment site (Fig. 2).

**Table 1**. Coordinates and features measured on the trees of the empirical landscape.

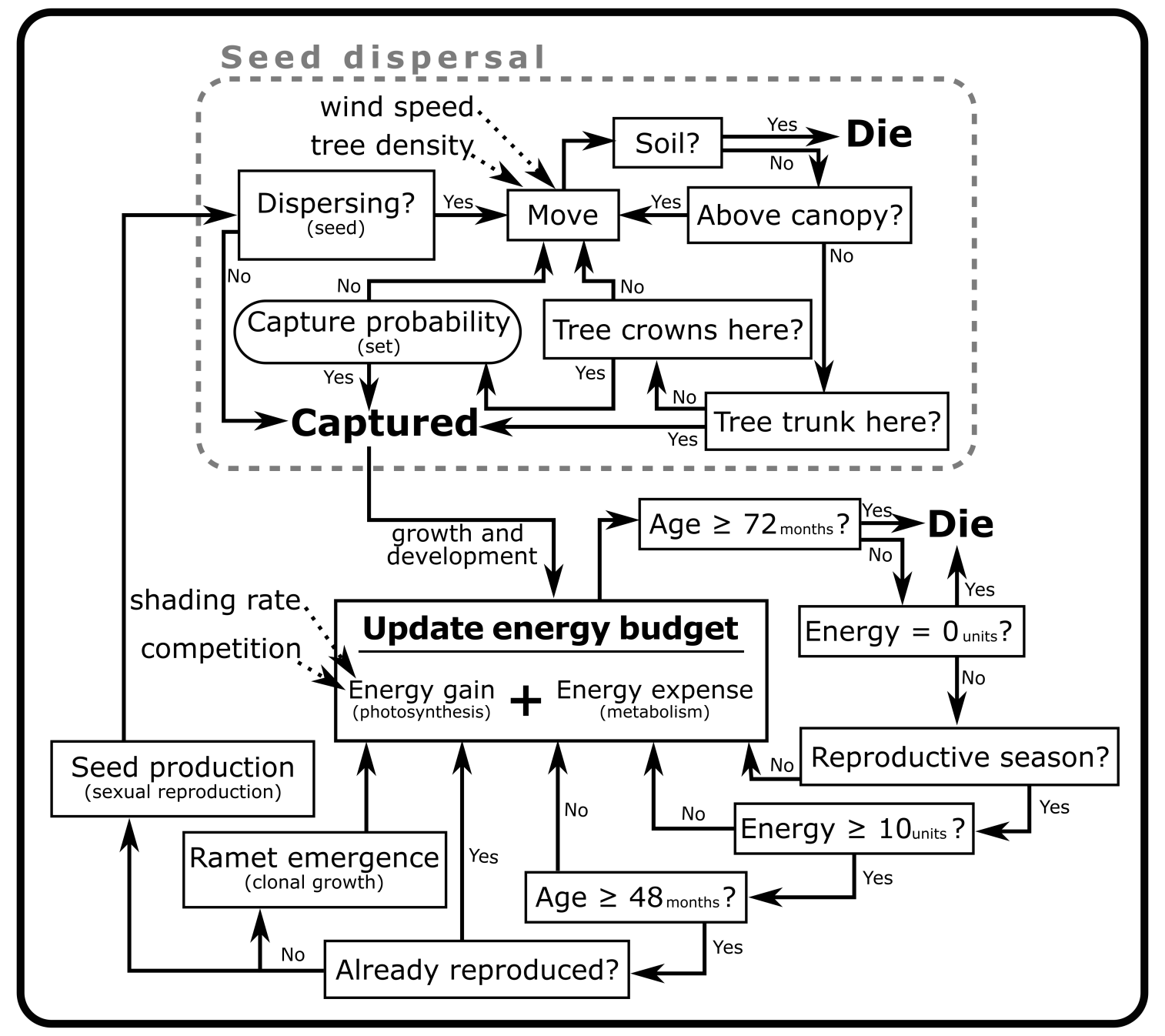


**Figure 2.** Flowchart of the main steps of the TRec model of the *Tillandsia recurvata* population spread. Dotted lines highlight the external local influence of landscape on the dispersal seed movement and on the individual energy budget. Units of energy are hypothetical.

1. *Process overview and scheduling*

Based on scientific literature and personal observations on the life cycle of *T. recurvata*, the following list of processes is executed once per time step (i.e. monthly):

1. **Update:** The TRec model updates the age in months of all *T. recurvata* individuals and calculates their energy budget (Fig. 2). The latter is grossly based on the gains and expenses by photosynthesis and metabolism (in hypothetical units) taking into account the shading rate, local space sharing, and individual age. If enabled, tree sizes are yearly updated according to their individual-specific growth rate, which is defined according to a normal distribution with a mean predefined value of tree-growth rate.
2. **Reproductive season:** Every year (i.e. corresponding to 12-time steps) the model adds new *T. recurvata* seeds to the system. These seeds come from outside of the system (hereafter referred to as 'regional seed rain') or from reproducing individuals already attached to trees of the system (hereafter referred to as 'local seed rain'). The regional seed rain is initially the main source of genetic diversity for subpopulations (i.e. individuals attached to the same tree), as each newly introduced seed has a random genotype where the alleles and heterozygosity of each locus follow the observed in the empirical data. The genotypes of seeds from local seed rain, in turn, are a combination of the maternal gametes' genotypes, which contributes to the increasing in the abundance of local multilocus genetic lineages (MLLs). Here, MLLs are referred to as a group of individuals in which the common ancestor is the first seed that arrived from the regional seed rain. Nevertheless, locally produced seeds can also increase the genetic diversity of a population, since they are subject to mutations by DNA replication slippage. Moreover, adult individuals also have clonal growth, producing new ramets that stay attached to them and have the same genotypes. To produce new seeds or grow by cloning, *T. recurvata* individuals have to have enough energy during the reproductive season. This energy is acquired throughout the year and is balanced with the energy spent on metabolism. For this reason, it is reduced in established individuals under heavy competition or strong tree crown overlapping (i.e. shading rate).
3. **Seed movement:** Dispersed seeds move straightforward in a random direction, gradually reducing their height. They can be captured if they are at the same height and location as a tree trunk or crown. If by chance, the seed is not captured by the trunks or crowns, its velocity reduces drastically, due to the weaker wind speed inside canopies (see Nathan et al. 2002). Seeds that reach the ground (height = 0) or to the margins of the simulated landscape are excluded from the model.
4. **Seed germination:** The model calculates the germination probability of seeds being captured by a tree trunk or crown. If not germinated, they are excluded from the model.
5. **Mortality:** If an individual reaches its lifespan age or if accumulated energy reaches zero, it is also excluded from the model.
6. **Landscape dynamics:** If enabled, it allows for arboreal growth and simulates landscapes with gradual and/or abrupt changes as follows:

6.1. **Gradual dynamics:** Landscapes gain or lose trees yearly by regeneration or self-thinning, respectively. The rate of each one depends on the number and size of pre-existing trees and, relative to self-thinning, to the rate of crown overlapping on each tree

6.2. **Abrupt dynamics:** After 30 years of simulation, landscapes can abruptly gain or lose trees according to predefined rates of reforestation and deforestation, respectively.

*4. Design Concepts*

- *Basic principles*

The TRec model assumes that similar to metapopulations models, *T. recurvata*'s populations on each tree can go extinct or be rescued due to distinct rates of seed dispersal that arise from their drought tolerance and their specific reproductive system that includes obligatory self-fertilization, due to cleistogamous flowers, and intense clonal growth. Under this view, *T. recurvata* has ‘closed MLLs’, where the genetic diversity in a population increases only by the arrival of seeds from a new lineage or by the mutation of locally produced offspring.

- *Emergence*

The genetic structure of the simulated *T. recurvata* population emerges from the seed dispersal processes that vary according to predefined parameters related to *T. recurvata* traits (seed capture probability, mutation rate) and landscape characteristics (wind speed, regional seed rain, tree density, and gradual and abrupt dynamics). It is measured with *a posteriori* analysis that uses the genotypes of up to 15 randomly sampled individuals composing each of the 14 subpopulations sampled in the empirical landscape or up to 15 subpopulations formed in simulated landscapes.

- *Stochasticity*

If the landscape is set as simulated, the position of trees is chosen randomly from the unoccupied patches. Regional seeds enter the landscape from a random point in its margins, or from points of the corresponding margin from which the direction of regional seed rain is set. Gradual and especially abrupt dynamics stochastically change the size and number of potential host trees for *T. recurvata*, which may lead to strong changes in the population of the simulated epiphyte.

- *Observation*

A 2D display produced in NetLogo software (version 6.1; Wilensky, 1999) allows for the monitoring of *T. recurvata* population spreading in the simulated landscape. It shows a top view looking down on a landscape formed by trees, represented by green circular crowns with a trunk show as a brown and smaller concentric circle. Moving colorful points represent dispersing *T. recurvata* seeds which develop into seedlings and adults when stop after being trapped by a tree trunk or crown. Distinct colors for each individual indicate distinct MLLs. Despite being displayed as a 2D reproduction, all simulations have a '3D behavior', once the model assigns distinct heights of each tree trunk, crown, and *T. recurvata* individual attachment. Therefore, each tree can be thought of as a disk-shaped crown with a cylindrical trunk attached underneath its core. Moreover, despite that the representation shows a 2D movement, each dispersed seed also changes its altitude at each movement step.

*5. Initialization and input data*

The simulation can start with (i) a representation of the empirical landscape; or with (ii) a simulated landscape. In the first case, 20 trees with specific heights of trunks and crowns are distributed over the area in specific positions (Table S1). In the second case, the set number of trees is distributed randomly over the available area with a previously set average crown area and hosting no *T. recurvata* individuals. All simulations are run in a homogeneous environment, where, beyond chosen wind speed, only the number of trees and their underlying crown overlapping affect the dispersal speed.

*6. Submodels*

In the following, details of the main processes are presented.

1. **Update**

Every time step (i.e. simulated month), the age and energy of all bromeliads are updated. The age is updated adding a month, and the energy is updated according to their energy budget that reduces the energy spent for metabolism from the energy produced through photosynthesis:

- Energy expense: the metabolism processes take 0.3 and 0.5 units of the accumulated energy of, respectively, adults (age > 48 months) and seedlings (age < 48 months). This difference is due to the greater energy spent by seedlings for their growth;
- Energy gain: photosynthesis adds a variable amount of energy, according to the following function:

(1)

energy gain = (((2-(1-periphery))/shading)/compet)

where *the periphery* is where in tree crown the individual is located (individual _altitude_/tree _height_), *shading* is the number of tree crowns that cover the individual’s location, and *compet* is the number of other adult individuals in a radius of 15 cm from the focal individual. If 'tree-growth' is enabled, every simulated tree in the model has an individual-specific growth rate which is defined according to a normal distribution with a predefined mean value. Such growth rate equally affects all dimensions of tree size (i.e. DBH, trunk height, crown height, and crown area).

1. **Reproductive season**

The reproductive season takes place every year (i.e. every 12-time steps representing one month each), where new individuals of *T. recurvata* are added from outside of the system (regional seed rain) or from other reproducing individuals in the system. Given *T. recurvata* is a monocarpic species (Mercier & Endres 1999), each individual ramet with at least 10 units of energy in the model can reproduce and produce new ramets by clonal growth only once during their life cycle. After reproduction, each individual loses two-thirds of its original energy.

*Regional seed rain*

A previous set number of regionally dispersed seeds are included in the simulation. Their alleles in each of the defined SSR loci are randomly chosen between the shortest and longest alleles found in the empiric study, following their motifs (i.e. dinucleotide or trinucleotide) and with the heterozygosity observed in the empirical study. The dispersal origin of these seeds could be previously defined as north, south, east, west, or random. Their starting height is randomly chosen from 1 meter to up to five meters more than the average height of trees in the simulated landscape. The color which can be observed in the 2D-display for each seed is chosen randomly and can be seen as a new MLL entering into the simulated landscape. If an individual reproduces during the simulation, its color is inherited by its offspring.

The regional seed rain can also be increased linearly every year throughout the simulation. The increment can be linear:

(2)

seed rain ^regional^ = seeds + (seeds * year)

where the parameter *seeds* are the previously set number of seeds in the regional seed rain. This increment mimics a growth of source populations outside the simulated landscape.

*Seed production*

The number of seeds that a reproducing individual produces is ten times its amount of energy. Each newly produced seed starts to move from the same location where it was produced in a random direction. As *T. recurvata* is a cleistogamous species, each produced seed has a genotype that is random recombination of the alleles of its mother (i.e. if the mother genotype for a locus is 202|208, the offspring genotypes will be the following: 202|202, 202|208, and 208|208). However, if the mutation option in the model is ‘on’, each allele of the new seeds genotypes can gain or lose repeat units by chance (i.e. Stepwise Mutation Model), simulating the process known as “DNA replication slippage” of microsatellites, in a rate that is also previously set in a range between 10^-2^ and 10^-6^. Therefore, in this model, the mutation is the single source of variation within MLLs. To track back MLLs, each new seed has a flag indicating the ID of the first individual of its MLL that arrived in the simulation.

*Clonal growth*

During the reproductive seasons, the same individuals that produced seeds also develop clonal growth, producing more ramets (hereafter referred to as 'clones') according to their energy (N_clones_ = energy/5). Half of the initial energy of the reproducing individual is shared equally among all new ramet. The clones stay attached close to their mother and have the same multi-locus genotype (MLG) and multi-locus lineage (MLL).

1. **Dispersal seed movement**

*move*

*T. recurvata* seeds are dispersed only by wind during the reproductive season (here delimited by a month within a year). Their movement is randomly divided into five ‘steps’, representing the distinct conditions they can face throughout the landscape. The wind speed is previously set by the user and is measured by an arbitrary metric that is related to the seed dispersal speed during the reproductive season. This metric represents how many model patches (0.01 m^2^) the seed moves at each step.

During a reproductive season, a dispersal seed moves straightforward, in a randomly chosen direction, losing altitude by chance. The distance the seed moves at each movement step depends on its position, above or below the average height of tree crowns within the ca. 3 meters of the radius. If the seed is above this threshold, its speed is five times the set wind speed and its altitude reduces 0.1 m at each movement step. However, if the seed is below this threshold, the wind speed effect on its movement decreases as the following function:

(3)

distance_step_ = speed_wind_ - (2 * shading - (0.1 * altitude))

where, *shading* is the number of canopies that cover the individual site, and *altitude* is the location of the dispersed seed in the air, measured from the ground. According to the above function, the number of tree crowns covering a site reduces the speed dispersal, but this effect is weakened by the seed's altitude inside the canopy. In other words, the higher a seed is in the canopy, and the fewer trees are in the landscape, the greater is the effect of wind on its dispersal. Seeds below the canopy threshold also decrease in altitude by 0.5 m at each movement step.

If a seed reaches a tree trunk, it will be captured, but, if it reaches a tree crown, the model calculates its capture probability. This probability is previously set in the model by the user (ranging from 0-100%) and it is multiplied by the number of tree crowns that cover the space. All seeds that reach the ground or the landscape margins, or that are not captured during the reproductive season, die before the next month.

1. **Seed germination and seedling survival**

From all seeds that enter the simulated landscape or are produced in it, the model calculates the germination probability that is previously set by the user (from 0 to 100%) and a 65 % of chance of seedling survival.

1. **Mortality**

An individual dies when its age reaches its lifespan limit of three years (i.e. 72 months), already reproduced once, or when its energy is lower or equal to zero. To reach energy equals zero, energy gain has to be lower than the expense. The energy of an individual can also be reduced in a stochastic event if it is activated in the model. If so, it can occur in a random month of each half time of the simulated period of 10 years. During these events, all bromeliads are affected losing 5-25 energy units.

1. **Landscape dynamics**

The model can be run with a simulated static landscape, when the number and size of all trees remain the same during the simulation procedure, or with a dynamic landscape. For the last option, four parameters change the number and the size of trees along with the simulations, thus, affecting the dynamics of *T. recurvata* populations. These parameters can be activated and set according to the user goals, and are divided as tree-growth (see the ‘Update’ topic above), gradual dynamic, and abrupt dynamics, as follows:

*Gradual dynamics*

- *Regeneration*: yearly, new trees are added to the landscape according to the set rate which takes into account 'adult' trees as the following equation:

N_newtrees_ = N_adulttrees_ * N_offspring_ * regeneration_rate

where N_newtrees_ is the number of new trees added, N_adulttrees_ is the number of adult trees (here, defined as trees with size ≥ 100), and N_offspring_ represents the number of offspring each tree will produce (here, a random number between 1 and 5). New trees are always included in areas with no other tree trunk or crown overlapping. They initially have the same size (DBH = 10 cm, trunk and crown height = 1 m), but the growth rate is randomly set from a normal distribution with the average value set by the user (see the ‘update’ topic above).

- *Self-thinning*: if activated in the simulation, self-thinning follows Yoda's law, which considers the average DBH and tree density in the landscape to forecast strong competition among trees that naturally reduces tree density. For this, in each simulated year, the model first calculates the DBH/tree-density as:

log(DBH_trees_) / (log(N_trees_ / 0.4) / 1.605)

where DBH_trees_ and N_trees_ are the average DBH and the number of trees with DBH > 50 cm. The self-thinning takes place when the general growth of trees overpasses the expected balance with tree density (i.e. if the DBH/tree-density ratio value is higher than 1). If so, trees overlapped by the crowns of at least two other larger trees are excluded from the model.

*Abrupt dynamics*

- *Reforestation*: the model includes the exact number of trees in the system as set by the reforestation_rate. As in the ‘Regeneration’ dynamic, new trees are always included in areas with no other tree trunk or crown overlapping. They initially have the same size (DBH = 10 cm, trunk and crown height = 1 m), but the growth rate is randomly set from a normal distribution with the average value set by the user (see the ‘update’ topic above).
- *Deforestation*: the model excludes random trees, according to the number of trees with DBH > 50 cm and the set deforestation_rate. All bromeliads hosted on those trees are also excluded from the model.

*7. References*

Benzing, D. H. (2000). *Bromeliaceae: profile of an adaptive radiation*. Cambridge: Cambridge University Press.

Benzing, D. H. (2012). *Air Plants: epiphytes and aerial gardens*. New York, NY: Cornell University Press.

Bernal, R., Valverde, T., & Hernandez-Rosas, L. (2005). Habitat preference of the epiphyte *Tillandsia recurvata* (Bromeliaceae) in a semi-desert environment in Central Mexico. *Botany*, 83(10), 1238-1247.

Birge, W. I. (1911). The anatomy and some biological aspects of the “ball moss”, tillandsia recurvata L. *Bulletin of The University of Texas*, *194*(20).

Chaves, C. J. N., Aoki-Gonçalves, F., Leal, B. S. S., Rossatto, D. R., & Palma-Silva, C. (2018). Transferability of nuclear microsatellite markers to the atmospheric bromeliads Tillandsia recurvata and T. aeranthos (Bromeliaceae). *Brazilian Journal of Botany*, (August). doi:10.1007/s40415-018-0494-4

Chilpa-Galván, N., Márquez-Guzmán, J., Zotz, G., Echevarría-Machado, I., Andrade, J. L., Espadas-Manrique, C., & Reyes-García, C. (2018). Seed traits favouring dispersal and establishment of six epiphytic Tillandsia (Bromeliaceae) species. *Seed Science Research*, 1–11. doi:10.1017/S0960258518000247

Flores-Palacios, A., García-Franco, J. G., & Capistrán-Barradas, A. (2015). Biomass, phorophyte specificity and distribution of Tillandsia recurvata in a tropical semi-desert environment (Chihuahuan Desert, Mexico). *Plant Ecology and Evolution*, *148*(1), 68–75.

GBIF Secretariat (2017). GBIF Backbone Taxonomy. Checklist dataset https://doi.org/10.15468/39omei accessed via GBIF.org on 2018-10-23.

McWilliams, E. (1992). *Chronology of the natural range expansion of Tillandsia recurvata (Bromeliaceae) in Texas*. *Contributions to Botany* (Vol. 15).

Mercier, H., Endres, L. (1999) Alteration of hormonal levels in a rootless epiphytic bromeliad in different phenological phases. *Journal of Plant Growth Regulation*, 18, 121-125.

Nathan, R., Horn, H. S., Chave, J., & Levin, S. A. (2002). Mechanistic models for tree seed dispersal by wind in dense forests and open landscapes. In *Seed Dispersal and frugivory-Ecologie, Evolution, Conservation* (pp. 69–82). doi:10.1079/9780851995250.0069

Orozco-Ibarrola, O. A., Flores-Hernández, P. S., Victoriano-Romero, E., Corona-López, A. M., & Flores-Palacios, A. (2015). Are breeding system and florivory associated with the abundance of Tillandsia species (Bromeliaceae)? *Botanical Journal of the Linnean Society*, *177*(1), 50–65. doi:10.1111/boj.12225

Smith, A. K., Martin, C. E., & Lüttge, U. (1989). Gas exchange and water vapor uptake in the atmospheric CAM bromeliad Tillandsia recurvata L.: the influence of trichomes. *Botanica Acta*, *102*, 80–84.

Smith, L. B., & Downs, R. J. (1977). Tillandsioideae (Bromeliaceae). In *Flora Neotropica* (pp. 661–1178).

Soltis, D. E., Gilmartin, A. J., Rieseberg, L., & Gardner, S. (1987). Genetic Variation in the Epiphytes Tillandsia ionantha and T. recurvata (Bromeliaceae). *American Journal of Botany*, *74*(4), 531–537.

Wilensky, U. (1999). NetLogo. http://ccl.northwestern.edu/netlogo/. Center for Connected Learning and Computer-Based Modeling, Northwestern University, Evanston, IL.
