# Supplementary material for: Deforestation is the turning point for the spreading of a weedy epiphyte: an IBM approach
Source: Sci Rep. 2021 Oct 14;11:20397. doi: 10.1038/s41598-021-99798-5 (PMC8516858; doi:10.1038/s41598-021-99798-5)
Supplement: Supplementary file 2 — Supplementary Information 2. [file 41598_2021_99798_MOESM2_ESM.doc]

**Table S1.** Sample number (N), number of alleles (A), allelic richness (A_R_), private alleles (A_P_), expected heterozygosity (H_E_), observed heterozygosity (H_O_), and fixation index (F_IS_) for each sampled population.

**
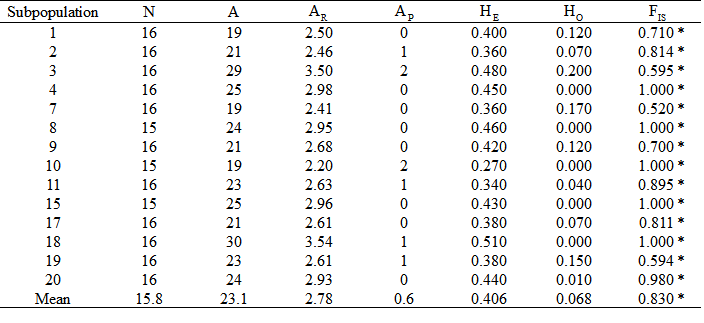
**

* Deviations from Hardy-Weinberg Equilibrium (P<0.05).


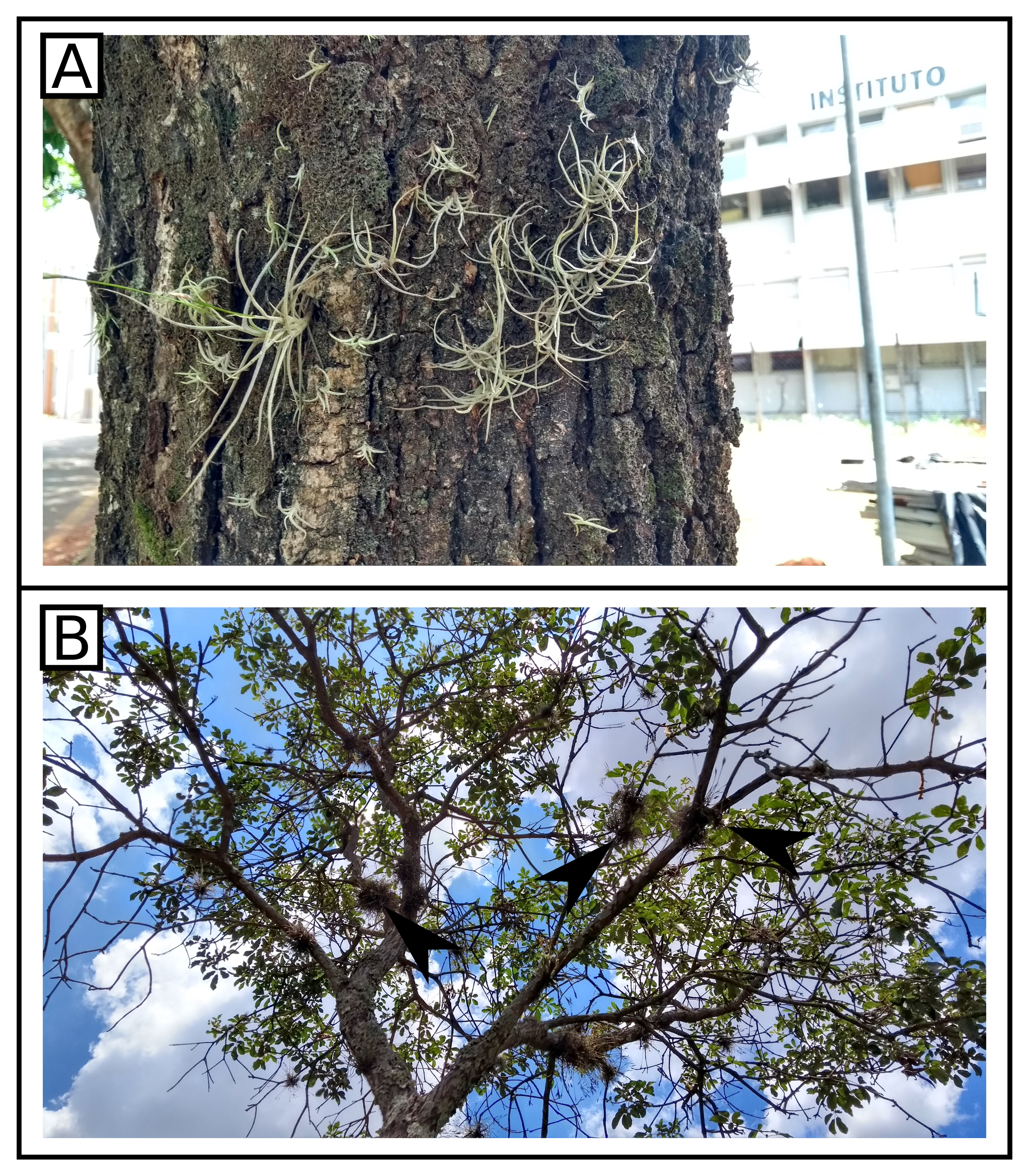


**Figure S1.** Photographs showing an adult and seedlings of *Tillandsia recurvata* (A), as well as the typical ‘ball shape’ of many (B) *T. recurvata* ramets groups (arrow heads).


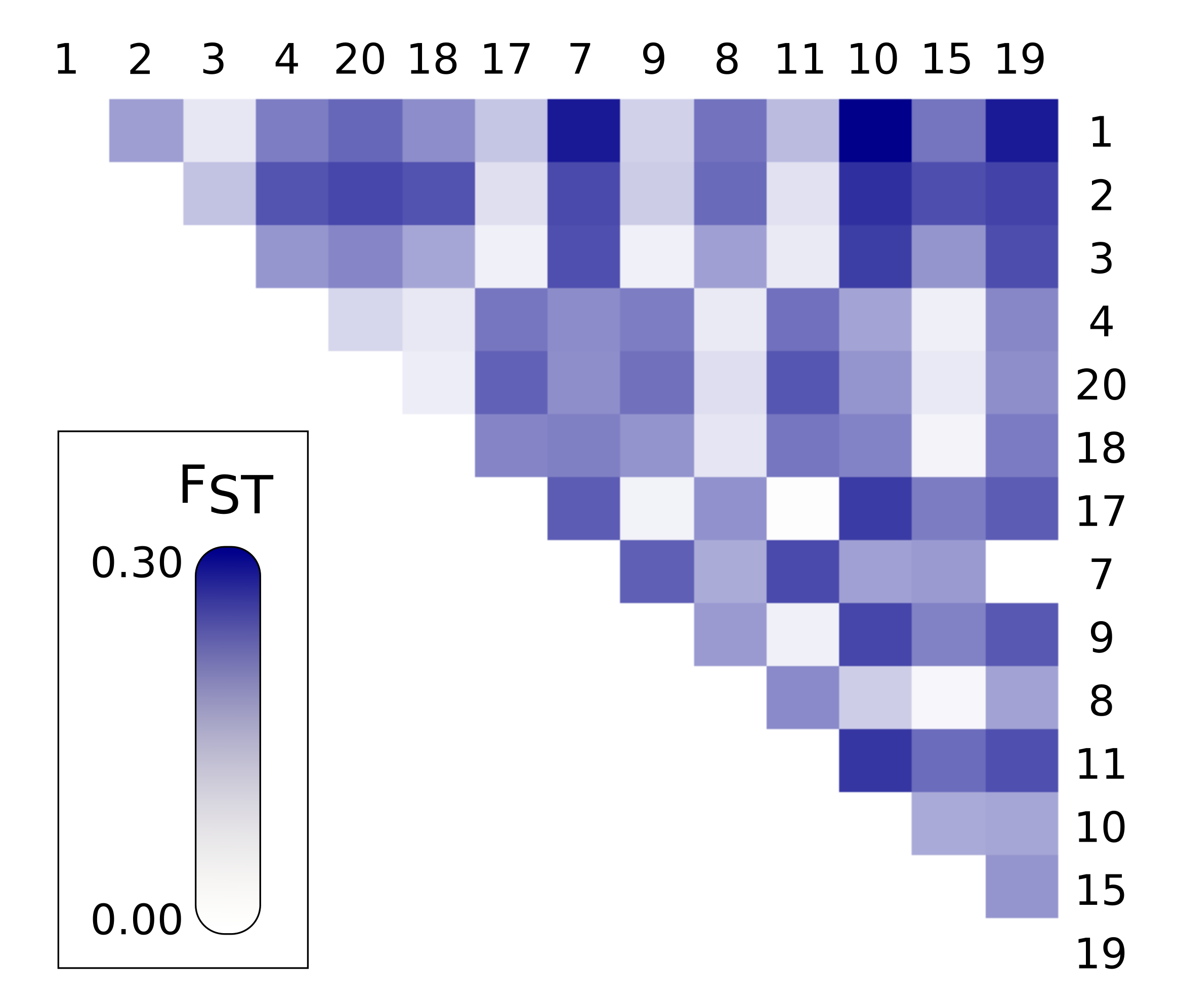


**Figure S2.** Squared matrix representation of subpopulation differentiation of *Tillandsia recurvata.*


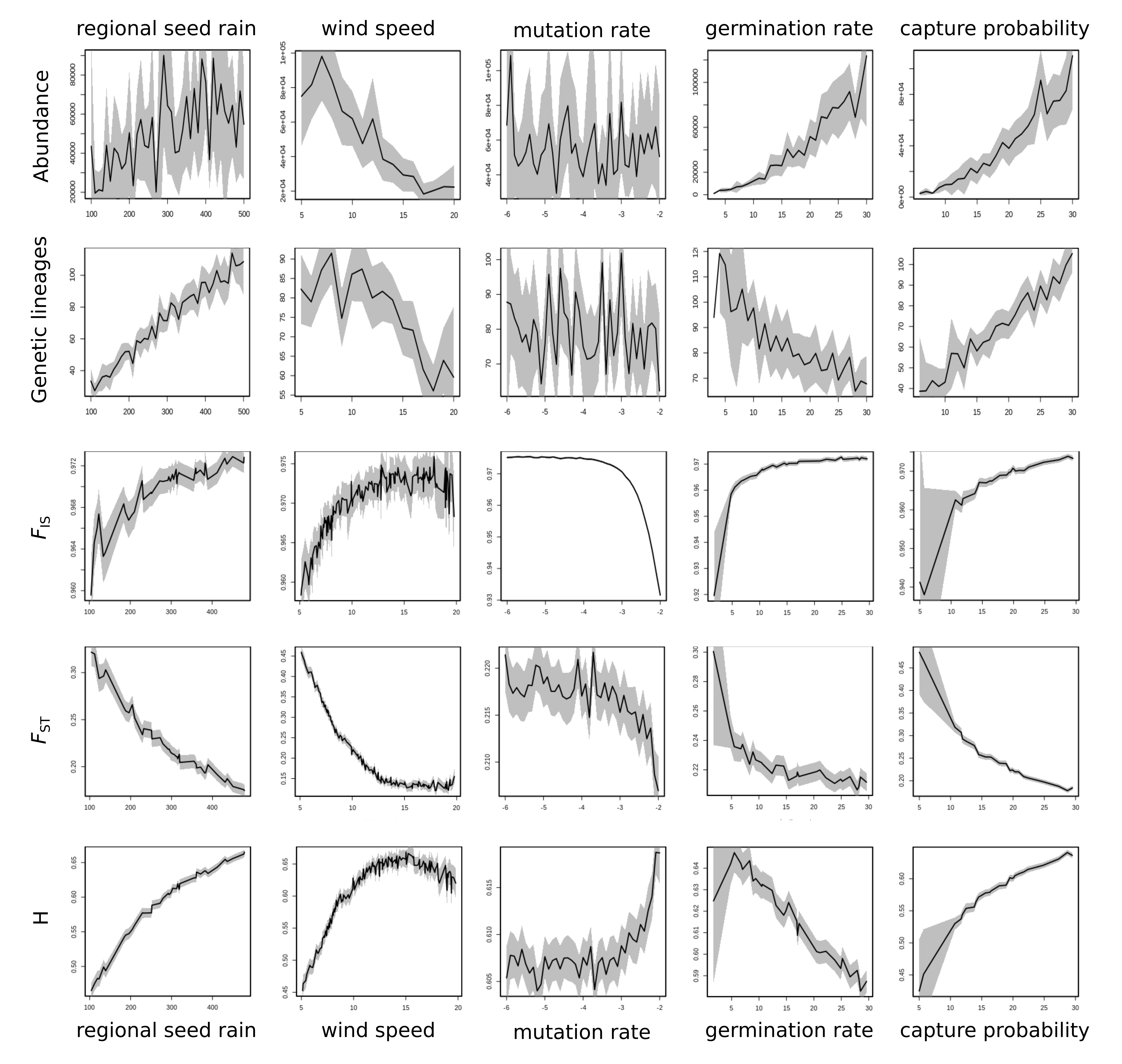
**Figure S3**. Sensitivity analysis of the IBM model. X-axes represent the parameters’ variation; Y-axes represent the summary statistics, as well as *T. recurvata* abundance and total number of genetic lineages. F_IS_: mean fixation index; F_ST_: mean population differentiation; He: mean expected heterozygosity.


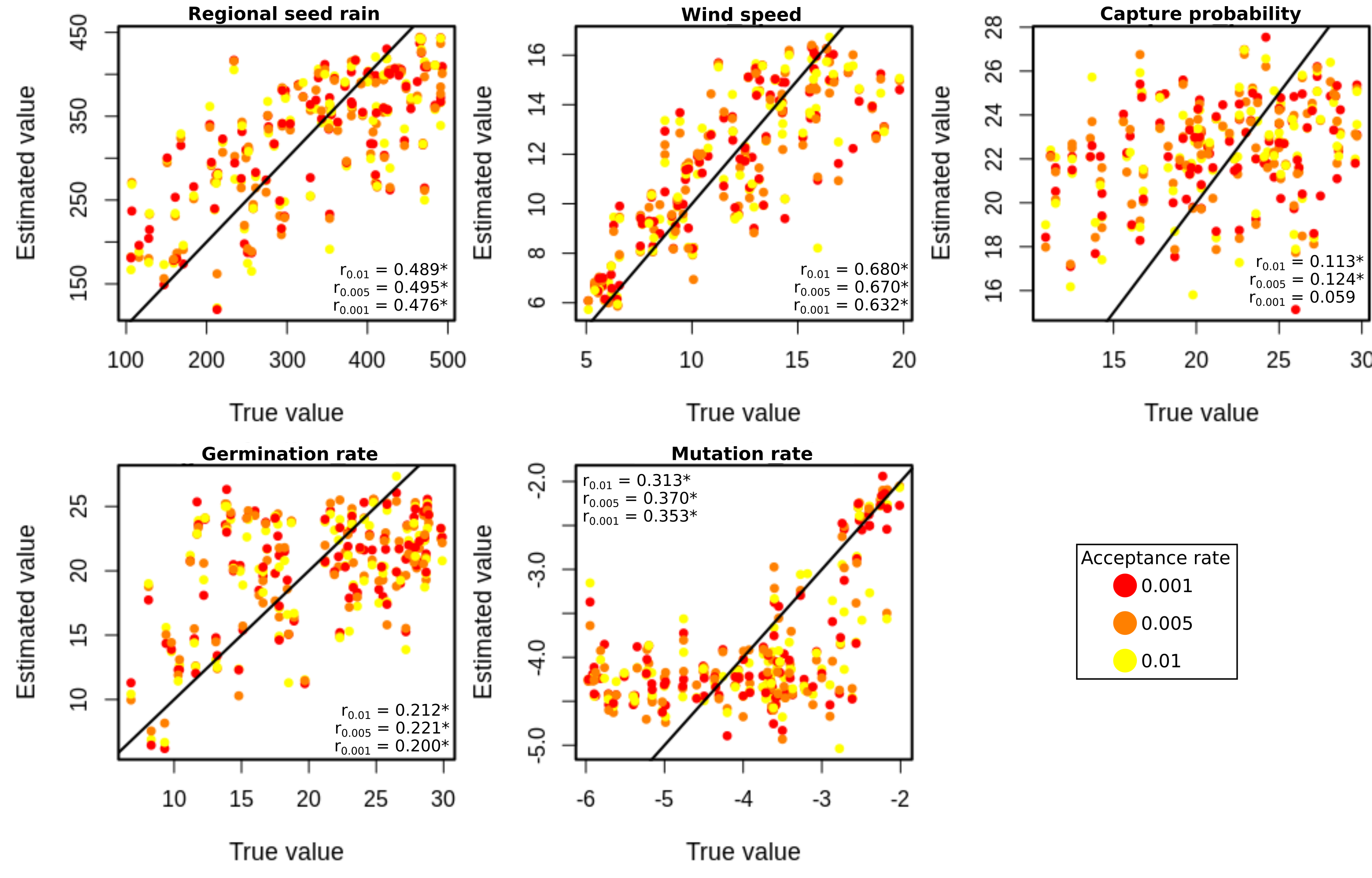
**Figure S4.** Cross-validation for parameter estimation. Parameter values estimated by ABC in relation to true values for each of the five IBM parameters. Spearman’s correlation coefficients (r) are shown at each panel for each acceptance rate.





**Figure S5.** The patterns obtained from multiple simulated spreading of *Tillandsia recurvata* populations in the empirical static landscape (see Fig. 1), along 50 years (A), and in static landscapes with distinct tree densities (B). Figures from the first to the last row show variances in: the inbreeding coefficient (F_IS_), mean expected heterozygosity (H_e_), turnover of multi-locus genotypes (T/O_MLG_) and multi-locus lineages (T/O_MLL_), and Sp statistics. Lines and yellowed areas around represent the average and 95 % confidence intervals of each variable in 2,500 (A) and 7,000 (B) simulations. In A, the stages of individual abundance development by time (i.e. “Lag”, “Log”, and “Stationary”) are delimited by vertical dotted lines. In B, vertical dotted lines highlight turning points in the individual abundance and FST of T. recurvata populations (D1-D4). Stars highlight the 4th simulated year, when the first arriving seeds reach to maturity. Down and up arrowheads highlight tree densities with the lowest and highest FST, respectively.


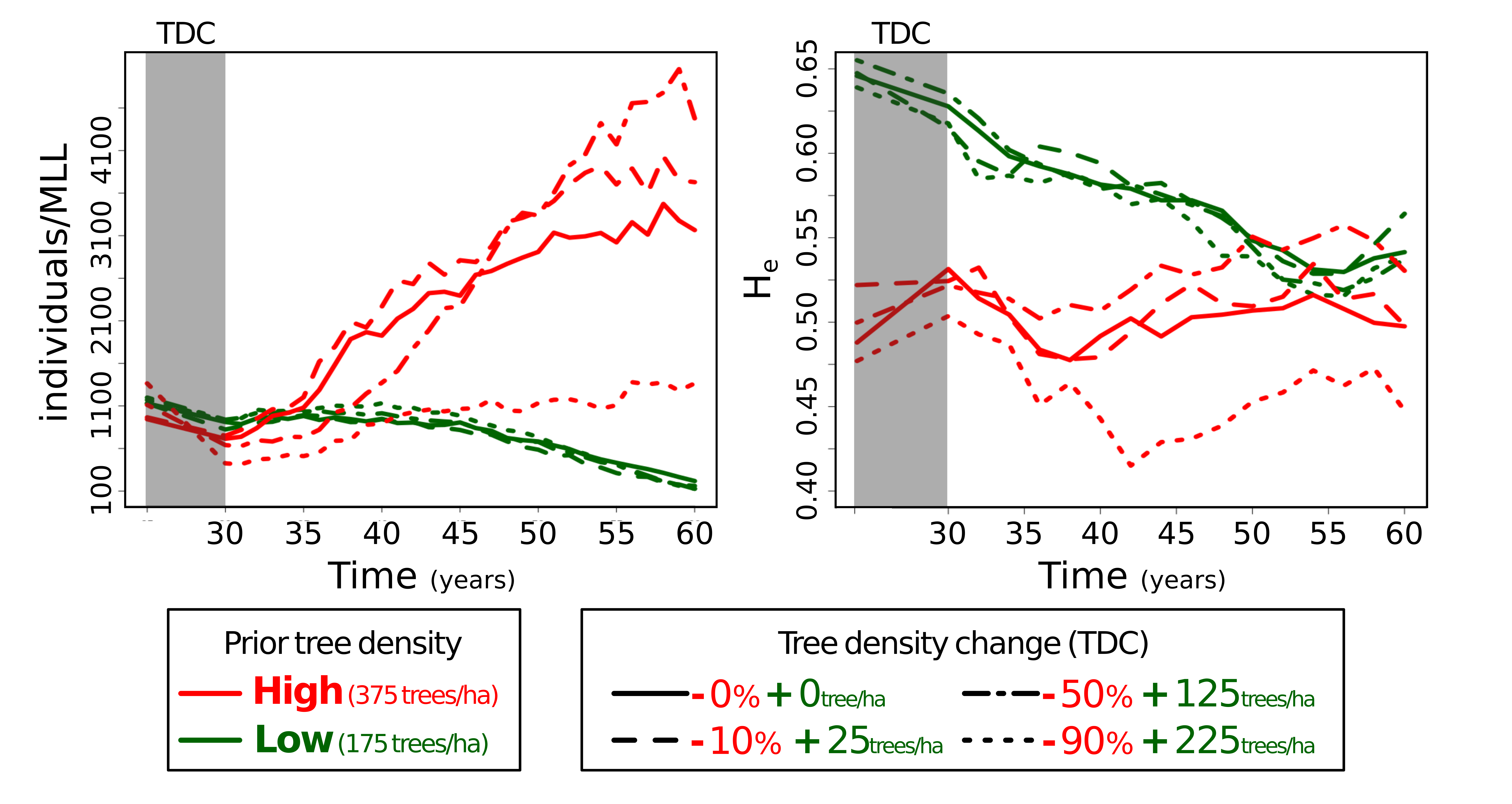


**Figure S6.** The temporal patterns obtained from multiple simulated spreading of *Tillandsia recurvata* populations in dynamic landscapes (emulating tree growth, regeneration, and self-thinning of natural forests) with prior high and low tree densities after abrupt reductions (lines in red) or increments (lines in green) in tree densities (gray rectangle). The first 30 simulated years, not showed here, correspond to the static simulations in landscapes presenting the lowest and highest FST (considering high tree density conditions) showed in Fig. 4 B. Tthe figures show 30-years of posterior temporal variation in the number of individuals by MLL and mean expected heterozygosity (H_e_) of T. recurvata’s populations. Lines represent the averages of each represented variable in 2,000 simulations. Solid, dashed, dotted-dashed, and dotted lines represent, respectively, absent, low, intermediate, and high anthropogenic changes in tree density (TDC).
